# Data-driven discovery of high performance layered van der Waals piezoelectric NbOI_2_

**DOI:** 10.1038/s41467-022-29495-y

**Published:** 2022-04-07

**Authors:** Yaze Wu, Ibrahim Abdelwahab, Ki Chang Kwon, Ivan Verzhbitskiy, Lin Wang, Weng Heng Liew, Kui Yao, Goki Eda, Kian Ping Loh, Lei Shen, Su Ying Quek

**Affiliations:** 1 Department of Physics, National University of Singapore, Singapore, Singapore; 2 Centre for Advanced 2D Materials and Graphene Research Centre, Singapore, Singapore; 3 Department of Chemistry, National University of Singapore, Singapore, Singapore; 4 Institute of Materials Research and Engineering, Agency for Science, Technology and Research (A*STAR), Singapore, Singapore; 5 NUS Graduate School, Integrative Sciences and Engineering Programme, National University of Singapore, Singapore, Singapore; 6 Department of Mechanical Engineering, National University of Singapore, Singapore, Singapore; 7 Engineering Science Programme, National University of Singapore, Singapore, Singapore; 8 Department of Materials Science and Engineering, National University of Singapore, Singapore, Singapore; 9 Present Address: Institute of High Performance Computing, Agency for Science, Technology and Research (A*STAR), Singapore, Singapore

**Keywords:** Two-dimensional materials, Electronic structure

## Abstract

Using high-throughput first-principles calculations to search for layered van der Waals materials with the largest piezoelectric stress coefficients, we discover NbOI_2_ to be the one among 2940 monolayers screened. The piezoelectric performance of NbOI_2_ is independent of thickness, and its electromechanical coupling factor of near unity is a hallmark of optimal interconversion between electrical and mechanical energy. Laser scanning vibrometer studies on bulk and few-layer NbOI_2_ crystals verify their huge piezoelectric responses, which exceed internal references such as In_2_Se_3_ and CuInP_2_S_6_. Furthermore, we provide insights into the atomic origins of anti-correlated piezoelectric and ferroelectric responses in NbOX_2_ (X = Cl, Br, I), based on bond covalency and structural distortions in these materials. Our discovery that NbOI_2_ has the largest piezoelectric stress coefficients among 2D materials calls for the development of NbOI_2_-based flexible nanoscale piezoelectric devices.

## Abstract (web summary)

The recent thrust toward flexible nanoscale devices creates a need for two-dimensional piezoelectric materials. Here, the authors find large piezoelectric response in NbOI_2_ flakes ranging from 4 nm to the bulk.

## Introduction

Piezoelectric materials enable the interconversion between mechanical and electrical energy. This is made possible by the change in polarization of the material when it is stretched or compressed. As such, piezoelectric materials are integral components of intelligent, multi-functional devices and drive a multi-billion dollar industry^1^ through their applications as sensors, actuators, energy harvesters, *etc*.^2–7^. The recent thrust toward flexible nanoscale devices creates a need for two-dimensional (2D) piezoelectric materials. Piezoelectric materials comprised of one or few layers of layered van der Waals (vdW) systems are particularly useful for increasingly important niche applications such as actuators with extreme atomic-scale precision^8^ as well as wearable sensors and smart material applications that require a large voltage signal in response to a small amount of physical deformation. 2D piezoelectric materials provide a practical alternative to micro-scale battery packs, functioning as nano-generators to power nanoscale devices^9^.

Thus far, the discovery of 2D piezoelectric materials has mostly been ad hoc, for example, by performing calculations on specific 2D materials that are known to be ferroelectric. However, with an ad hoc approach, it is difficult to ascertain if the 2D material indeed has optimal piezoelectric coefficients. Experimentally, it is also challenging to quantitatively compare the piezoelectric coefficients of 2D materials^10^. The objective of this work is to perform a systematic high throughput search through a 2D material database, in order to rank the 2D materials according to the size of their intrinsic piezoelectric coefficients. While 2D materials down to nanometers in thickness are sufficient for flexible nanoscale devices, symmetry-breaking in the monolayer can lead to the emergence of piezoelectricity in the monolayer even when the parent bulk materials are not piezoelectric^11^. Thus, we focus our search on monolayers. Out of 109 piezoelectric monolayers that we identify, the family of niobium oxydihalides NbOX_2_ (X = Cl, Br, I) is predicted to have among the largest in-plane piezoelectric stress coefficients, an order of magnitude larger than those of most reported 2D materials. We note that NbOX_2_ has recently been independently identified to be a robust room temperature ferroelectric in another high-throughput study searching for 2D ferroelectric materials^12^. While all ferroelectric materials are piezoelectric, there is no direct correlation between the magnitude of spontaneous polarization $\left|\vec{P}\right|$ and the magnitude of piezoelectric coefficients (see Supplementary Fig. S1). Within the NbOX_2_ family, our calculations in fact show that the piezoelectric and ferroelectric effects have opposing trends down the halogen group. We further show that the large piezoelectric effect is independent of crystal thickness, in contrast to MoS_2_ and similar 2D in-plane piezoelectrics, where the piezoelectricity vanishes for an even number of layers^13–15^. This thickness-independent piezoelectric effect is a practical advantage in isolating 2D nanoscale piezoelectrics. Experimental validations of the piezoelectric effect were carried out on few-layer NbOI_2_ and NbOCl_2_ crystals, where significantly larger piezoelectric coefficients were obtained compared to internal references such as In_2_Se_3_ and CuInP_2_S_6_ (known 2D piezoelectrics)^16–20^. Our findings pave the way for the development of NbOI_2_-based flexible nanoscale piezoelectric devices, such as high precision actuators and wearable electronics or energy-harvesters.

## Results and discussion

**Fig. 1:**
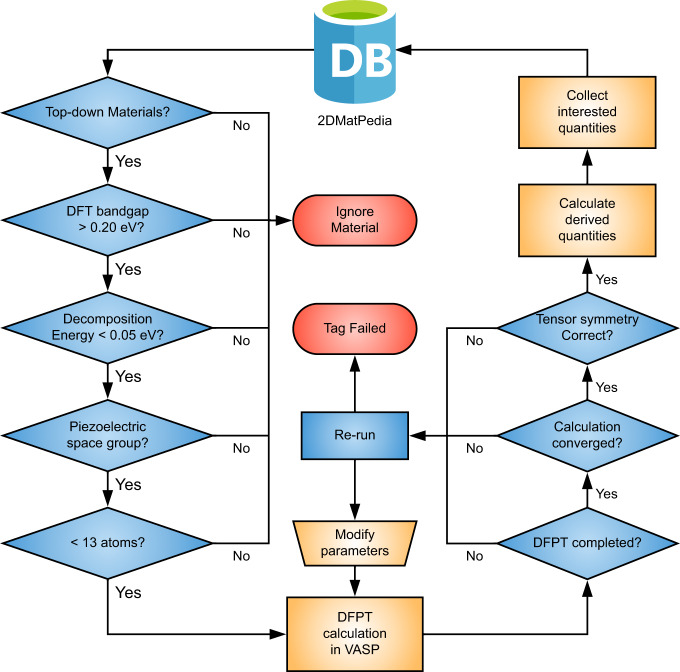
Workflow of the high-throughput calculation to screen for piezoelectric 2D materials. We have chosen a criteria of at least 0.20 eV for the DFT band gap (large enough for operation at finite temperatures) and a decomposition energy^21^ of < 0.05 eV to ensure thermodynamic stability.

The workflow of our high-throughput calculations is shown in Fig. 1. Our results are publicly available in 2DMatpedia^21^, an open database of 2D materials that shares the same infrastructure and basic workflow as the Materials Project database^22^. We focus only on the 2940 “top-down” materials within the database, which are obtained by exfoliation of known bulk layered materials, and are more likely to be dynamically stable and experimentally available. Next, we perform a rapid screening process based on the band gap and decomposition energy (both documented in 2DMatpedia^21^) as well as the space group (piezoelectric space groups lack inversion symmetry). A total of 225 materials pass this screening process. We then limit our high-throughput density functional perturbation theory (DFPT) calculations to materials with less than 13 atoms per unit cell (160 of the 225 materials). Following conventions for 2D materials, we compute the sheet piezoelectric stress tensor elements, ***e***_*ij*_, defined as $\frac{\partial {{{{{{\boldsymbol{P}}}}}}}_{i}}{\partial {{{{{{\boldsymbol{\eta }}}}}}}_{j}}L$, the rate of change in polarization ***P***_*i*_ with homogeneous strain ***η***_*j*_ multiplied by the cell height ***L***^23^. The index *i* runs from 1 to 3 (*x*, *y*, *z*) and *j* ranges from 1 to 6 (*xx*, *yy*, *zz*, *yz*, *xz*, *xy*) where the Voigt notation is used. A series of automated checks and analyses is carried out and the relevant data is saved into the database. 51 materials did not pass the automated checks. This is similar in proportion to those in other high throughput studies^1,24,25^; these materials were not studied in detail. The dynamical stability of individual materials is checked manually as needed outside this workflow.

**Fig. 2:**
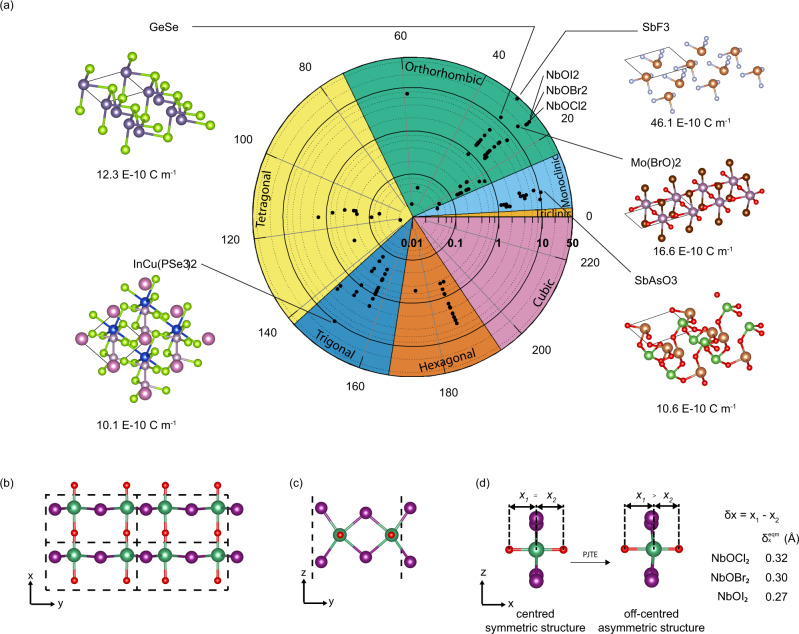
Maximum sheet piezoelectric tensor elements and atomic structures of selected materials. **a** High-throughput calculation results for maximum sheet piezoelectric stress tensor elements (***e***_*ij*_). The radial axis represents the magnitude of ***e***_*ij*_ in units of 10^−10^ C m^−1^ on a log scale and the angular axis represents the 230 space groups. Materials with ***e***_*ij*_ larger than 10 × 10^−10^ C m^−1^ are labeled and their atomic structures are presented. The maximum *e*_*ij*_ corresponds to ***e***_26_ for SbF_3_ and to ***e***_11_ for NbOX_2_. Atomic structures of selected materials are also presented. **b** Top view and **c** Side view down the *x*-axis to the atomic structure of monolayer NbOX_**2**_ (X = I, Br, Cl). Dark green balls denote Nb atoms; purple balls denote halogens; red balls denote O atoms. Dashed lines mark the unit cell boundaries. **d** Schematic, viewed along *y*-axis, showing relative displacement of Nb atoms along the *x* -axis away from the high symmetry position where $\delta x=0$ Å, to the equilibrium position where $\delta x={\delta }_{x}^{{eqm}}$. Additional structural parameters are provided in Supplementary Table S3. PJTE refers to the pseudo-Jahn-Teller effect.

The final results are summarized in Fig. 2a. All the 109 materials (see Supplementary Table S15 for the full list) have an ***e***_*ij*_ value greater than 0.05 × 10^−10^ C m^−1^. These 109 2D piezoelectric materials are observed to belong to only a few space groups (Fig. 2a). Space groups 17, 26, 31, 149, 156, and 187 each have more than nine 2D piezoelectric materials. Our high throughput calculations also found that 48 of the 109 2D piezoelectric materials are not ferroelectric (see Supplementary Table S15). 2D materials previously identified to be piezoelectric^23,26^ are also found to be piezoelectric in our calculations, with values of ***e***_*ij*_ very close to their reported values (Supplementary Table S2). Most materials have maximum ***e***_*ij*_ values below 5 × 10^−10^ C m^−1^, while a few have significantly larger ***e***_*ij*_. These larger ***e***_*ij*_ values correspond to in-plane piezoelectricity.

We identify eight materials with maximum sheet ***e***_*ij*_ values larger than 10 × 10^−10^ C m^−1^, namely, SbF_3_, NbOI_2_, NbOBr_2_, NbOCl_2_, MoBr_2_O_2_, GeSe, SbAsO_3_, CuInP_2_Se_6_. The structures of these eight materials (referred to as the ‘top 8’ materials) are presented in Fig. 2a–d. SbF_3_, which has the largest sheet ***e***_*ij*_, is found to be dynamically unstable in the monolayer form. Niobium oxydihalides, NbOX_2_, with X = Cl, Br, or I, have the next highest sheet ***e***_*ij*_, with NbOI_2_ having the largest ***e***_11_ value of ~32 × 10^−10^ C m^−1^. We have verified explicitly that monolayer NbOI_2_ is dynamically stable (Supplementary Fig. S2 and Fig. S3) and we expect the same to be true for the other members of the family. The exfoliation energy for NbOI_2_ monolayers is 18.2 meV Å^− 2^, lower than that of graphene (25.5 meV Å^−2^)^21^.

Besides the piezoelectric stress tensor elements ***e***_*ij*_, the piezoelectric strain tensor elements ***d***_*ij*_ are also widely discussed in the literature. The ***d***_*i*_ values are defined as $\frac{\partial {{{{{{\boldsymbol{P}}}}}}}_{i}}{\partial {{{{{{\boldsymbol{\sigma }}}}}}}_{j}}$, the rate of change in polarization ***P***_*i*_ with homogeneous stress ***σ***_*j*_ (see SI), and are related to ***e***_*ij*_ according to ${{{{{{\boldsymbol{d}}}}}}}_{{ij}}={{{{{{\boldsymbol{e}}}}}}}_{{ik}}{{{{{{\boldsymbol{S}}}}}}}_{{kj}}$, where ***S***_*kj*_ is the compliance tensor (the inverse of the elastic tensor). Unlike ***e***_*ij*_ and ***S***_*kj*_ which have a different definition for the 2D case, the ***d***_*ij*_ values are defined in the same manner for both bulk and 2D (see Formalisms in SI). The ***d***_11_ values for monolayer NbOX_2_ are ~42, 30 and 27 pm V^−1^ for NbOI_2_, NbOBr_2_ and NbOCl_2_, respectively (see Supplementary Table S4). We note that all the ***d***_*ij*_ values for bulk NbOX_2_ are almost the same as for the monolayer (Table 2 and Supplementary Table S5), indicating that the piezoelectric properties of NbOX_2_ are very similar from monolayer to bulk form. In the bulk material, the ratio of mechanical stress energy density to the electrical energy density is given by a dimensionless number ${k}^{2}=\frac{{{{{{{\boldsymbol{e}}}}}}}_{{ij}}{{{{{{\boldsymbol{d}}}}}}}_{{ij}}}{{{{{{{\boldsymbol{\varepsilon }}}}}}}_{{ij}}{\varepsilon }_{0}}$^27,28^, where *k* is known as the electromechanical coupling factor. We obtain an electromechanical coupling factor of ~1.0, 0.9 and 0.9 for bulk NbOI_2_, NbOBr_2_ and NbOCl_2_, respectively (see Supplementary Table S5). Since the maximum value of *k* is unity, we see that the intrinsic piezoelectricity in NbOX_2_ provides for highly efficient interconversion between electrical and mechanical energy.

**Fig. 3:**
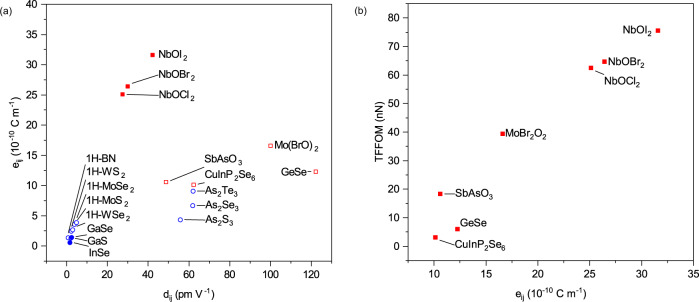
Thin film figure of merit (TFFOM) and spontaneous polarizations. **a** Plot of maximum sheet piezoelectric stress tensor elements (***e***_*ij*_) and corresponding piezoelectric strain tensor elements (***d***_*ij*_) for various 2D materials. For additional notes on GeSe, please see Supplementary Table S1. **b** Plot of TFFOM and maximum ***e***_*ij*_ of materials highlighted in Fig. 2**a**. In **a**, solid symbols denote materials that are piezoelectric in the thermodynamically most stable bulk form, as documented in the Materials Project database^1^; hollow symbols denote those that are not. Blue dots denote data points obtained from other studies^17,23,26,29,49^ listed in Supplementary Table S2. Our computed ***e***_*ij*_ values for these materials are also provided in Supplementary Table S2 for comparison.

We compute the ***d***_*ij*_ values for the top 8 materials (excluding SbF_3_) and the largest components of ***e***_*ij*_ and ***d***_*ij*_ are shown in Fig. 3a and Table 1. The maximum ***e***_*ij*_ and ***d***_*ij*_ values for other 2D piezoelectrics discussed in the literature are also plotted in Fig. 3a for comparison (see also Supplementary Table S2). Some materials such as monolayer GeSe^29^, As_2_S_3_^17^, As_2_Se_3_^17^ have large ***d***_*ij*_ values but small ***e***_*ij*_ values, corresponding to small values of their Young’s moduli. The small Young’s moduli limits the amount of force exerted in electric field-induced deformations. The 2D Young’s moduli C_11_ for monolayer NbOI_2_ is ~76 N m^−1^ (Supplementary Table S7) while the bulk value for C_11_ is ~125 GPa. A figure of merit adopted for thin-film piezoelectrics (TFFOM), when the passive elastic layer is much thicker than the piezoelectric material, is $\frac{{{{{{{\boldsymbol{e}}}}}}}_{{ij}}^{2}}{{{{{{{\boldsymbol{\varepsilon }}}}}}}_{{ij}}{\varepsilon }_{0}}$, where ${{{{{{\boldsymbol{\varepsilon }}}}}}}_{{ij}}$ is the dielectric constant and ${{{{{{\boldsymbol{\varepsilon }}}}}}}_{0}$ is the vacuum permittivity^27,30,31^. Thus, the piezoelectric stress coefficients rather than the piezoelectric strain coefficients are particularly important for 2D flexible piezoelectric applications.

**Table 1:** Materials with *e*_*ij*_ > 10 × 10^−10^ C m^−1^.

Material	ij	*e*_*ij*_ (10^−10^ C m^−1^)	*d*_*ij*_ (pm V^−1^)	*C*_*ij*_ (N m^−1^)	TFFOM (nN)
NbOI_2_	11	31.6	42.2	75.6	71.7
NbOBr_2_	11	26.4	30.0	89.0	63.2
NbOCl_2_	11	25.1	27.4	92.9	59.6
MoBr_2_O_2_	22	16.6	100.1	33.8	39.3
GeSe	22	12.3	122.2	18.2	6.0
SbAsO_3_	11	10.6	48.7	22.3	18.4
CuInP_2_Se_6_	22	10.1	62.3	44.5	3.1

***e***_*ij*_ is the piezoelectric stress tensor element, ***d***_*ij*_, is the piezoelectric strain tensor element, ***C***_*ij*_, is the elastic tensor element, and TFFOM is the figure of merit for thin film piezoelectric devices.^27^ Our computed in-plane dielectric constant of NbOX_2_ is 12–15 (see Supplementary Table S6).

It is clear that NbOX_2_ has the largest ***e***_*ij*_ values in Fig. 3a. To our knowledge, there is only one 2D material, SnSe, with a predicted ***e***_11_ value^29^ that is larger than NbOI_2_. Recent experiments on SnSe have, however, reported a much weaker piezoelectric performance^9^ (see Supplementary Table S8), which we attribute to the Poisson effect, which reduces the effective ***e***_11_ value of SnSe by ~51% (see Supplementary Table S1). We note that our high throughput calculations do not account for the Poisson effect. However, the Poisson effect does not change the ***e***_11_ values for NbOX_2_ (see Supplementary Table S1). The figure of merit (TFFOM) for our top 8 candidates from the high throughput calculations (except SbF_3_) are presented in Fig. 3b and Table 1, where it is clear that NbOI_2_ has the largest TFFOM.

In Fig. 3a, we also indicate using solid symbols the materials that are piezoelectric in the thermodynamically most stable bulk form, and hollow symbols for those that are not. NbOX_2_ are among the minority of 2D materials that are piezoelectric both in the monolayer and in the bulk. The ***d***_*ij*_ values are essentially the same for both monolayer and bulk NbOX_2_ (see Table S9). Of the 109 piezoelectric materials that we discovered, only 30 are also piezoelectric in the bulk, according to a similar high throughput study^1^ on the Materials Project database for bulk materials, from which the candidate monolayers were derived. A comparison of the largest ***e***_*ij*_ values from the two independent studies indicates that the piezoelectric coefficients in the monolayer and the bulk are strongly correlated (see Supplementary Table S10 and Supplementary Fig. S4). The thickness-independent piezoelectric effect in NbOX_2_ implies that few-layer NbOX_2_ can be prepared for nanoscale piezoelectric applications without the need for a pre-selection process based on the number of layers.

The atomic structure of monolayer NbOX_2_ is shown in Fig. 2b, c. Along the *y-*direction, Peierls distortion results in alternating Nb-Nb distances, and along the *x-*direction, the Nb atom is displaced away from the high symmetry position where *δx* = *x*_1_–*x*_2_ (Fig. 2d), giving rise to a spontaneous polarization (Table 2). The degree of structural asymmetry along the *x-*direction is largest for NbOCl_2_ and smallest for NbOI_2_ (Fig. 2d). The asymmetry along the *x-*direction can be explained by the pseudo-Jahn-Teller effect (PJTE)^32^, where mixing between the valence O *p* orbitals and conduction Nb *d* orbitals results in a more energetically favorable configuration accompanied by structural distortion as well as increased Nb-O bond strengths and covalency (see SI for details). The piezoelectric tensor elements in Table 2 reflect the strong in-plane anisotropy of the system. In bulk NbOX_2_, the directions for the inversion-symmetry-breaking distortions are the same in all layers, and the difference in ${\delta }_{x}^{{eqm}}$ is within 0.002 $\text{\AA}$ in the bulk and monolayer systems (Supplementary Table S3 and Table S11). Thus, the piezoelectric properties are similar in bulk, monolayer and thin film form.

**Table 2:** Piezoelectric tensor elements and spontaneous polarizations of NbOX_2_.

Formula	*e*_11_	*e*_12_	*e*_13_	*e*_26_	*e*_35_	*d*_11_	*d*_12_	*d*_26_	*P*_*x*_
NbOCl_2_	25.1	−1.1	−0.4	0.8	0.0	27.4	−4.1	5.4	185
NbOBr_2_	26.4	−1.0	−0.4	0.8	0.0	30.0	−4.1	5.8	170
NbOI_2_	31.6	−1.0	−0.3	0.7	0.0	42.2	−5.1	5.2	143

Sheet piezoelectric stress tensor elements ***e***_*ij*_ are in units of 10^−10^ C m^−1^ and piezoelectric strain tensor elements ***d***_*ij*_ are in pm V^−1^. Piezoelectric tensor elements that are zero due to symmetry of the space group are omitted here. Spontaneous polarization along the *x-*direction (***P***_*x*_) in pC m^−1^ is calculated as the difference between the polarization of the equilibrium and symmetric structures.

Quantitative measurements of piezoelectric coefficients in 2D materials are challenging, since any small parasitic vibration, boundary effect, or electrostatic force during the piezoelectric measurement (especially the single-point measurement) significantly affects the accuracy of the measured values^10^. To provide quantitative information about the piezoelectric coefficients within the family of NbOX_2_, and to compare the piezoelectric coefficients with those of other known 2D piezoelectrics, α-In_2_Se_3_ and CuInP_2_S_6_, we performed laser scanning vibrometer (LSV)^33^ measurements on thick bulk-like samples. All these materials are also piezoelectric in the bulk. The existence of ferroelectricity and piezoelectricity in thin films of NbOX_2_ was demonstrated using piezoresponse force microscopy (PFM) for thicknesses down to sub-10 nm.

We synthesize large-sized NbOI_2_ and NbOCl_2_ crystals grown by the chemical vapor transport method (refer to Methods for details). The crystal structures of the as-grown NbOI_2_ and NbOCl_2_ crystals are confirmed by single-crystal X-ray diffraction (SC-XRD) (Supplementary CIFs and Supplementary Table S11). The crystallographic directions of the NbOX_2_ crystals are also identified from the SC-XRD analysis. These room-temperature SC-XRD studies show that the crystal belongs to polar space group C2 (No. 5), hence providing further evidence that the ground state of bulk NbOX_2_ is ferroelectric (refer to Supplementary Note 1: Polarization switching in NbOX_2_ and Supplementary Note 2: Ferroelectric-paraelectric phase transition in NbOI_2_ for ferroelectric data).

To investigate if ultrathin NbOX_2_ is ferroelectric, nanosheets were exfoliated from bulk crystals via the Scotch tape method and then transferred onto gold (Au) substrates (Supplementary Fig. S5) for PFM characterization. NbOX_2_ is thermodynamically stable and all experimental measurements were performed under ambient conditions. In PFM, an AC voltage is applied between a conductive sharp tip and the bottom electrode of a piezoelectric sample to induce local mechanical deformations by means of the converse piezoelectric effect^34,35^. Scanning tip-induced hysteretic switching events were recorded using spectroscopic PFM (Supplementary Note 1: Polarization switching in NbOX_2_, and Fig. S19). The ferroelectric-paraelectric phase transition in NbOI_2_ was further observed using temperature-dependent differential scanning calorimetry and second harmonic generation measurements (Supplementary Note 2: Ferroelectric-paraelectric phase transition in NbOI_2_, and Fig. S20).

**Fig. 4:**
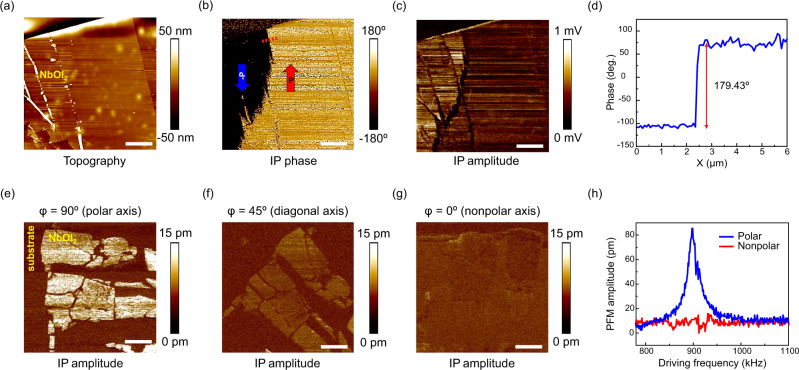
Piezoelectric force microscopy (PFM) investigation of thin NbOX_2_. **a** Topography, **b** In-plane (IP) phase, **c** IP amplitude, and **d** phase profile across antiparallel polarization states of 82-nm-thick NbOI_2_ flake. **e**–**g** Vector PFM IP amplitude images of 10-nm-thick NbOI_2_ showing spontaneous polarization at 90° **e**, 45° **f**, and 0° **g** angles relative to the cantilever long axis. **h** PFM amplitude profiles along the polar and nonpolar axes of the 10-nm-thick NbOI_2_ flake. Scale bars: 4 μm. Drive voltage: **b**, **c** 5 V, **e**–**h** 3 V. Drive frequency: **b**, **c** 65 kHz, **e** 827 kHz, **f** 974 kHz, **g** 890 kHz.

Figure 4a presents an 82-nm-thick NbOI_2_ flake with corresponding phase (Fig. 4b) and amplitude (Fig. 4c) images constructed using an in-plane (IP) PFM output channel. A clear contrast exists between different domains in the lateral phase and amplitude. The PFM phase indicates the direction of the ferroelectric polarization, whereas the PFM amplitude reflects the magnitude of the local piezoelectric response. The bright and dark contrasts in the PFM phase image indicate that there exist two oppositely polarized ferroelectric domains, characterized by a ~180° phase difference (Fig. 4d). Supplementary Fig. S6–S10 display additional PFM data for NbOI_2_ and NbOCl_2_, demonstrating piezoelectric response down to 4.3 nm-thickness for NbOI_2_.

To confirm the in-plane piezoelectric anisotropy, vector PFM^36,37^ is conducted on 10-nm-thick NbOI_2_ (Fig. 4e–h) and 17-nm-thick NbOCl_2_ (Supplementary Fig. S11) flakes with height profiles shown in Supplementary Fig. S12. As expected, the in-plane PFM response is strongest when the polar axis is orthogonal to the cantilever long axis, as shown in Fig. 4e (φ is the polarization angle relative to the cantilever long axis). When the sample is rotated by 90 degrees, no lateral PFM contrast is observed (Fig. 4g, h). In addition, the out-of-plane piezoresponses for the NbOX_2_ nanoflakes are typically negligible, as displayed in Supplementary Fig. S6 and Figs. S8–S10. The vector PFM results as well as predominantly in-plane response confirms that the physical origin of the PFM signals is piezoelectricity rather than electrostatic tip-sample interactions^38,39^.

**Fig. 5:**
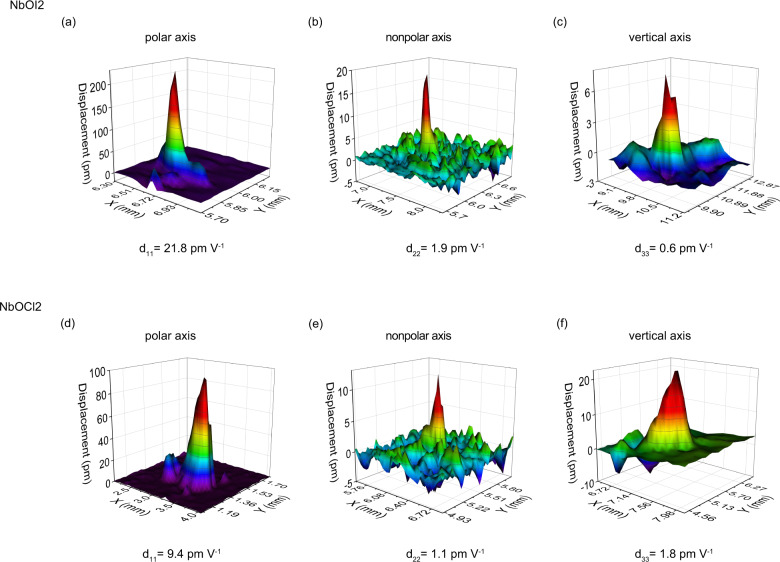
Evaluation of the piezoelectric coefficients of NbOX_2_ using a laser scanning vibrometer (LSV). 3D graphs of the instantaneous vibration when the displacement magnitude reaches the maximum under the sine-wave driving electrical signal. **a**, **d** Measurement along the lateral polar direction (***d***_11_). **b**, **e** Measurements along the lateral nonpolar direction (***d***_22_). **c**, **f** Measurements along the vertical nonpolar direction (***d***_33_).

We quantified the lateral (***d***_11_, ***d***_22_) and vertical (***d***_33_) piezoelectric coefficients of NbOX_2_ through laser scanning vibrometer (LSV)^33^ measurements. LSV is a non-contact optical technique that measures the vibration velocity of a moving piezoelectric surface by monitoring the interference pattern (Doppler frequency shift) between the scattered light and the incident light. By integrating the vibration velocity, the mechanical displacement in response to an applied electric field can be determined. The LSV vibration modalities are measured along the x (***d***_11_) (Fig. 5a, d), y (***d***_22_) (Fig. 5b, e) and z (***d***_33_) (Fig. 5c, f) directions of NbOX_2_. Consistent with the vector PFM data, the LSV vibration modalities further confirm the anisotropy of the piezoelectric response in NbOX_2_. Our measurements reveal that NbOI_2_ exhibits stronger piezoelectric effects than NbOCl_2_ (the ***d***_11(*eff*)_ values are ~21.8 pm V^−1^ for NbOI_2_ and ~ 9.4 pm V^−1^ for NbOCl_2_), consistent with our theoretical predictions (Table 2). These values are likely to be underestimated due to the significant electric leakage of the NbOX_2_ samples. Despite this, our measured ***d***_11(*eff*)_ piezoelectric coefficients are larger than the largest ***d***_*ij*(*eff*)_ values we measured for common 2D ferroelectrics, such as α-In_2_Se_3_ and CuInP_2_S_6_ (Supplementary Fig. S13). There is also a strong correlation between the measured ***d***_*ij*(*eff*)_ values and the corresponding computed values (Supplementary Fig. S14). These LSV results show that NbOX_2_ has superior piezoelectric performance compared to other 2D material piezoelectrics.

We analyze the origins of the large piezoelectric and ferroelectric effects in NbOX_2_, and their trends down the halogen group. The large values of ***e***_11_ arise predominantly from the lattice response to the applied strain (see Supplementary Table S12). This dominant ionic contribution can be written in an implied sum notation as

1 $${{{{{{\boldsymbol{e}}}}}}}_{{ij}}^{{ion}}={{{{{{\boldsymbol{Z}}}}}}}_{m,i}^{{{{{{\boldsymbol{* }}}}}}}\frac{\partial {{{{{{\boldsymbol{u}}}}}}}_{m}}{\partial {{{{{{\boldsymbol{\eta }}}}}}}_{j}}$$

where ${{{{{{\boldsymbol{Z}}}}}}}_{m,i}^{{{{{{\boldsymbol{* }}}}}}}$ is the dynamical charge (*m* is a composite label for atom and displacement direction and *i* is the direction of the polarization), ***u*** is the position vector of the atom and ***η***_*j*_ denotes the applied strain. The dynamical charge is defined as the rate of change of polarization with atomic displacement, while $\frac{\partial {{{{{{\boldsymbol{u}}}}}}}_{m}}{\partial {{{{{{\boldsymbol{\eta }}}}}}}_{j}}$ quantifies the rate of change in atomic displacement with applied strain. The dynamical charges play an important role in both the piezoelectric and ferroelectric effects.

**Fig. 6:**
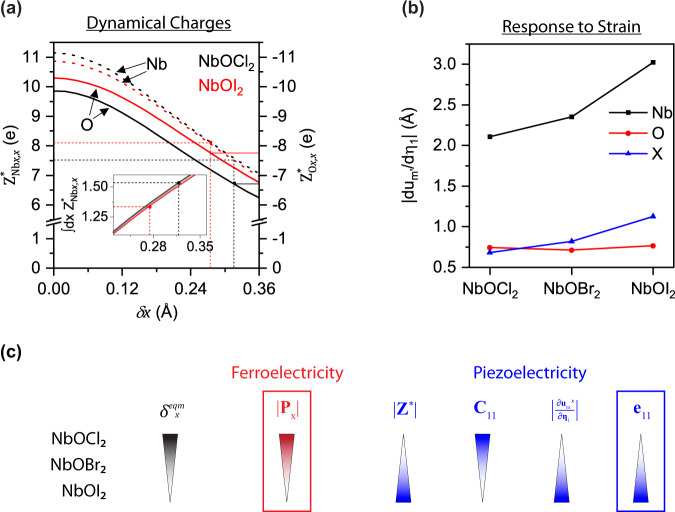
Understanding the origin of piezoelectric and ferroelectric effects in the NbOX_2_ family. **a** Dynamical charges for Nb and O, ${{{{{{\boldsymbol{Z}}}}}}}_{{Nbx},x}^{{{{{{\boldsymbol{* }}}}}}}$ and ${{{{{{\boldsymbol{Z}}}}}}}_{{Ox},x}^{{{{{{\boldsymbol{* }}}}}}}$. as a function of *δx*, the difference between the longer and shorter Nb-O bonds. The inset shows the integral of ${{{{{{\boldsymbol{Z}}}}}}}_{{Nbx},x}^{{{{{{\boldsymbol{* }}}}}}}$ with respect to the *x*-displacement away from the high-symmetry site. In both the main figure and the inset, dashed lines indicate the values in the equilibrium structures. **b**$\left|\frac{\partial {{{{{{\boldsymbol{u}}}}}}}_{{{{{{{\boldsymbol{m}}}}}}}^{{{{{{\boldsymbol{x}}}}}}}}}{\partial {{{{{{\boldsymbol{\eta }}}}}}}_{1}}\right|$ in the equilibrium structures of NbOX_2_, indicating the rate of change of *x-*displacement of each atom (Nb, O, or X) with strain in the *x-*direction. NbOI_2_ has the largest lattice response to strain, due to the smaller stiffness tensor elements and weaker Nb-O bonds. **c** Schematic illustrating the origins of the trends in ferroelectricity and piezoelectricity in NbOX_2_.

Figure 6a shows that the dynamical charges in the *x*-direction for Nb and O in NbOCl_2_ and NbOI_2_, decrease in magnitude as the off-center displacement, *δx*, increases. These dynamical charges are significantly larger in magnitude than their expected formal oxidation states (+4 for Nb and -2 for O) as well as their estimated static charges (see Supplementary Fig. S15). The anomalous dynamical charges can be attributed to the partial covalency present in the Nb-O bonds, which is more significant in NbOI_2_ due to the smaller electronegativity of I compared to Cl (see SI and Supplementary Fig. S16)^40^. The smaller off-center distortion in NbOI_2_ (smallest ${\delta }_{x}^{{eqm}}$) further increases the dynamical charges at equilibrium compared to those for NbOCl_2_, contributing to the superior piezoelectric performance of NbOI_2_. On the other hand, the integral of the dynamical charges with respect to atomic displacements, from the centered symmetric structure to the equilibrium off-centered structure, gives the magnitude of the spontaneous polarization at equilibrium, and the larger ${\delta }_{x}^{{eqm}}$ for NbOCl_2_ results in a larger $\left|\vec{P}\right|$ compared to NbOI_2_ (Fig. 6a inset).

The larger magnitudes of $\frac{\partial {{{{{{\boldsymbol{u}}}}}}}_{{m}^{x}}}{\partial {{{{{{\boldsymbol{\eta }}}}}}}_{1}}$ (the superscript for *m* representing displacements in the *x*-direction) for Nb and X atoms in NbOI_2_ (Fig. 6b) further contribute to the large ***e***_11_ value for NbOI_2_ (see also Supplementary Table S13). The observed trend in $\frac{\partial {{{{{{\boldsymbol{u}}}}}}}_{{m}^{x}}}{\partial {{{{{{\boldsymbol{\eta }}}}}}}_{1}}$ for Nb and X can be traced to the degree of bond covalency/ionicity in these systems, the more ionic NbOCl_2_ having stiffer bonds and hence smaller magnitudes of $\frac{\partial {{{{{{\boldsymbol{u}}}}}}}_{m}}{\partial {{{{{{\boldsymbol{\eta }}}}}}}_{j}}$. This trend can also be observed in the decrease in Young’s modulus ***C***_*11*_ down the halogen group (see Supplementary Table S7). Figure 6c summarizes the different factors that contribute to ***e***_11_ being largest for NbOI_2_, in contrast to $\left|\vec{P}\right|$ being largest for NbOCl_2_.

While our top candidates for monolayer piezoelectrics all exhibit in-plane piezoelectricity, we comment that out-of-plane piezoelectricity (non-zero ***e***_3*j*_) in the 2D monolayers is found in 46 of the materials in our database, as shown in Supplementary Fig. S17. Most of the non-zero ***e***_3*j*_ values have magnitudes less than 1 × 10^−10^ C m^−1^, with a few exceptions having values up to ~4 × 10^−10^ C m^−1^, as indicated in Supplementary Fig. S17 and Supplementary Table S14. It has been reported that bulk layered CuInP_2_S_6_ has an electromechanical coupling factor of 0.7–0.9, corresponding to a large out-of-plane piezoelectric coefficient which originates from the deformation of the van der Waals gap^41^. Such a mechanism has not been considered in our study on monolayers. Our findings that monolayer and bulk NbOI_2_ have exceptionally large in-plane piezoelectric effects are especially important for actuator applications that require pure in-plane movement^42^.

In summary, we have identified, from among 2940 candidate monolayers, NbOI_2_ as the material with the largest piezoelectric stress coefficient. Furthermore, NbOI_2_ is one of the minority of monolayer piezoelectrics that is also piezoelectric in the bulk. Our experimentally measured values of piezoelectric strain coefficients are within a factor of two of the predicted value for NbOI_2_, and are much larger than those measured concurrently for α-In_2_Se_3_ and CuInP_2_S_6_. We have also verified in-plane ferroelectricity in NbOI_2_. While NbOI_2_ has the largest piezoelectric coefficients, NbOCl_2_ has the largest spontaneous polarization. The excellent piezoelectric and ferroelectric effects in NbOX_2_, as well as their trends down the halogen group, are rationalized on the basis of bond covalency and symmetry-breaking structural distortions in these materials. The structure-property correlations obtained here provide guidance for the design of functional piezoelectric and ferroelectric materials, while the discovery of 2D NbOI_2_ as a high-performance piezoelectric paves the way for 2D piezoelectric devices.

## Methods

### Computational methods

For the high-throughput DFPT calculations, only materials from space groups 1, 3–9, 16–46, 75–82, 89–122, 143–146, 149–161, 168–174, 177–190, 195–199, 207–220^1^ are selected. These space groups lack inversion symmetry. DFPT calculations are performed with the plane-wave pseudopotential code VASP^43,44^, employing the generalized gradient approximation (GGA) for the exchange-correlation functional^45^. We use an energy cutoff of 520 eV, a Monkhorst-Pack k-point mesh with a density of 1500 per reciprocal atom (number of atoms per cell multiplied by the number of k-points)^1^, a force convergence criterion of 0.005 eV Å^−1^ and a criterion of 10^−10^ eV for the convergence of the self-consistent cycle. The calculations are performed with a vacuum separation of about 20 Å between the 2D materials.

We modify the standard workflows^46,47^ developed by Materials Project^1,22^ to include additional post processing steps that convert the 3D piezoelectric tensor elements to 2D sheet piezoelectric tensor elements in the unit of C m^−1^ by multiplying the former with the cell height. Other results, including the dynamical charge tensor (also known as the Born effective charge tensor), dielectric tensor (ionic and electronic contributions), Γ point phonon eigenvalue and eigenvectors, full piezoelectric tensor (in both C m^−2^ and C m^−1^), maximum (sheet) piezoelectric tensor elements in C m^−2^ (C m^−1^) and maximum (sheet) out-of-plane piezoelectric tensor elements in C m^−2^ (C m^−1^) are also captured. Our workflow also inherits the consistency checks and filters in the Materials Project Workflow^1^ to detect errors arising from DFT calculation and convergence-related issues.

For the targeted study on NbOX_2_, Monkhorst-Pack k point meshes of 12 × 6 × 1 including the Γ point are used. The atomic coordinates are fully relaxed using the conjugate gradient scheme until the maximum energy difference between iterations is less than 10^−8^ eV and the residual force is less than 0.001 eV Å^−1^. Other parameters are inherited from the high-throughput calculations.

For calculations with fixed *δx*, the *x*-coordinates of the atomic positions are fixed while the *y* and *z* coordinates are allowed to relax. The lattice constants are kept fixed. $\frac{\partial {{{{{{\boldsymbol{u}}}}}}}_{{{{{{{\boldsymbol{m}}}}}}}^{{{{{{\boldsymbol{x}}}}}}}}}{\partial {{{{{{\boldsymbol{\eta }}}}}}}_{1}}$ is obtained by applying strain in the *x*-direction and allowing the atoms to relax.

The elastic properties of NbOX_2_ are obtained using a finite difference method as implemented in VASP. The elastic tensors are adapted for 2D materials according to the treatment by Choudhary et al.^48^.

The piezoelectric strain tensor (***d***_*ij*_) is calculated from piezoelectric stress tensor (***e***_*ij*_) through Supplementary Equation S7. Elements in ***S***_*ij*_ related to the *z*-direction are set to 0.

### Synthesis of single crystals of NbOI_2_ and NbOCl_2_

Crystalline NbOI_2_ and NbOCl_2_ are grown by the chemical vapor transport method. High-purity Nb (film), iodine (crystals), and Nb_2_O_5_ (powder) with a stoichiometric ratio Nb:O:I = 1:1:2 are used as precursors for the growth of NbOI_2_. The mixture of precursors is placed in a quartz ampule, and the ampule is sealed after being evacuated (10^−5^ Torr). Similarly, Nb, NbCl_5_ (powder), and Nb_2_O_5_ are used to grow NbOCl_2_. The sealed quartz ampules are placed at the center of a horizontal dual-zone furnace. Both heating zones were slowly heated to 600 °C and holding for 5 days. The ampules are then slowly cooled for 10 days with slightly different rates at the hot (1.2 °C h^−1^) and cold (1.5 °C h^−1^) zones. After the slow-cooling process, the furnace is turned off allowing the ampules to cool down naturally. Crystals are extracted from the opened ampules under inert conditions of an N_2_-filled glove box and then stored for future use.

### X-ray diffraction (XRD) measurements

Single crystal X-ray diffractions of bulk NbOCl_2_ and NbOI_2_ crystals are measured using a four circles goniometer Kappa geometry, Bruker AXS D8 Venture, equipped with a Photon 100 CMOS active pixel sensor detector. A molybdenum monochromatized (λ = 0.71073 Å) X-Ray radiation is used for the measurement. The frames are integrated with the Bruker SAINT software using a narrow-frame algorithm. Data are corrected for absorption effects using the Multi-Scan method (SADABS). The structures are solved in the monoclinic unit cell and refined using the SHELXT, VERSION 2014/5 Software. The final anisotropic refinement of the structures is performed by least squares procedures on weighted F^2^ values using the SHELXL-2014/7 (Sheldrick, 2014) included in the APEX3 v2016, 9.0, AXS Bruker program.

### Piezoresponse force microscopy (PFM) characterization

Mechanical exfoliation is performed by peeling off as-grown crystals using the Scotch tape method. Exfoliated crystals are directly transferred onto fresh gold (Au) substrates for PFM characterization. PFM images are obtained using a Bruker Dimension Icon AFM in contact mode. Pt/Ir-coated silicon tips with a radius of 20 nm and a force constant of 0.4 N m^−1^ are used for the PFM measurements. The drive frequency and drive amplitude for the PFM images are ~30–975 kHz and 2.5–10 V, respectively. The PFM amplitude is expressed in pm or mV units depending on the selected PFM output channel. Angular-resolved vector PFM was performed by rotating the NbOX_2_ samples with respect to the cantilever axis. The spectroscopic PFM hysteresis loops were acquired with ±10 V DC sweeps while applying an AC voltage of 5 V. PFM was operated in various contact scanning modes to measure both in-plane (buckling and twisting/torsional modes) and out-of-plane (deflection mode) components of the piezoresponse independently and simultaneously.

### Laser scanning vibrometer (LSV) measurements

The effective piezoelectric coefficients of NbOX_2_, α-In_2_Se_3_, and CuInP_2_S_6_ (CIPS) are measured with a laser scanning vibrometer (OFV- 3001-SF6, PolyTech GmbH) after the crystals are DC poled under 150 V along the relevant crystallographic direction for 5 min. The LSV data are collected along the *x* (d_11_), *y* (d_22_) and *z* (d_33_) directions of NbOX_2_ and along the *z* (d_33_) directions of α-In_2_Se_3_ and CIPS under a unipolar AC signal of amplitude 10 V at 8 kHz through silver electrodes. The α-In_2_Se_3_ and CuInP_2_S_6_ crystals were purchased from HQGraphene. The effective piezoelectric coefficients are deduced from the profile analysis of the instantaneous displacement data to determine the strain generated under the sine-wave driving electrical signal.

### Ferroelectric Measurements

Polarization−electric field (P–E) loops were recorded using a ferroelectric tester (Precision Multiferroic II, Radiant Technologies). Lateral NbOI_2_ devices are fabricated by transferring NbOI_2_ flakes onto interdigitated platinum (Pt) electrodes (10/5 µm, electrode/gap) via poly (dimethyl siloxane) (PDMS) stamp-transfer method. The Pt contacts (70 nm thick) are patterned on thermally grown silicon dioxide using electron beam lithography, electron beam evaporation, and lift-off process. A transfer station with an optical microscope and a rotating stage is used to transfer the NbOI_2_ flakes in such a way that the device’s lateral electrical field is aligned along either the in–plane polar or nonpolar axis of the transferred flakes. The P–E loop is acquired with ±10 V sweeps.

Differential scanning calorimetry (DSC) analysis was performed under a nitrogen atmosphere with a heating rate of 10 °C min^−1^ using Mettler-Toledo DSC.

### Reporting summary

Further information on research design is available in the Nature Research Reporting Summary linked to this article.

## Supplementary information

Supplementary Information

Supplementary Dataset 1

Supplementary Dataset 2

Reporting Summary

## Data Availability

The datasets generated during and/or analyzed during the current study are available from the corresponding author on reasonable request.

## Code availability

The code used in this work is modified from the open-source tools developed by Materials Project, in particular, Python Materials Genomics (http://pymatgen.org/) and Atomate (https://atomate.org). The versions used in this work are pymatgen/2018.8.10 and atomate/0.8.2 for all the calculations.
