# Supplementary material for: Data-driven discovery of high performance layered van der Waals piezoelectric NbOI2
Source: Nat Commun. 2022 Apr 7;13:1884. doi: 10.1038/s41467-022-29495-y (PMC8990070; doi:10.1038/s41467-022-29495-y)
Supplement: Supplementary file 1 — Supplementary Information [file 41467_2022_29495_MOESM1_ESM.pdf]

SUPPLEMENTARY INFORMATION

Data-driven discovery of high performance layered van der Waals piezoelectric $NbOI_2$

Yaze Wu[#,1,2], Ibrahim Abdelwahab[#,2,3], Ki Chang Kwon[3], Ivan Verzhbitskiy[1,2], Lin Wang[3], Weng Heng Liew[4], Kui Yao[4], Goki Eda[1,2], Kian Ping Loh[2,3,5*], Lei Shen[6,7*], Su Ying Quek[1,2,5,8*]

[#]These authors contributed equally: Yaze Wu, Ibrahim Abdelwahab.

[1]Department of Physics, National University of Singapore, Singapore, Singapore.

Current address: Institute of High Performance Computing (IHPC) – Agency for Science, Technology and Research (A*STAR), 1 Fusionopolis Way, #16-16 Connexis, Singapore 138632

[2]Centre for Advanced 2D Materials and Graphene Research Centre, Singapore, Singapore.

[3]Department of Chemistry, National University of Singapore, Singapore, Singapore.

[4]Institute of Materials Research and Engineering, A*STAR (Agency for Science, Technology and Research), Singapore.

[5]NUS Graduate School, Integrative Sciences and Engineering Programme, National University of Singapore, Singapore.

[6]Department of Mechanical Engineering, National University of Singapore, Singapore, Singapore.

[7]Engineering Science Programme, National University of Singapore, Singapore, Singapore.

[8]Department of Materials Science and Engineering, National University of Singapore, Singapore.

*e-mail: chmlohkp@nus.edu.sg; shenlei@nus.edu.sg; phyqsy@nus.edu.sg

Table of Contents

Fig. S1 Plot of spontaneous polarization ($pC\ m^{-1}$) and maximum sheet e_{ij} for various 2D materials. ---------- 4

Table S1 Values of relaxed-ion $\boldsymbol{e}_{ij}$ ($10^{-10}\ C\ m^{-1}$) under different definitions. -- 4

Table S2 Relaxed-ion piezoelectric tensor element ($\boldsymbol{e}_{ij}$), piezoelectric strain tensor element ($\boldsymbol{d}_{ij}$), and spontaneous polarization ($\boldsymbol{P}_i$) of selected materials in units of $10^{-10}\ C\ m^{-1}$, $pm\ V^{-1}$ and $pC\ m^{-1}$ respectively. ---------- 5

Fig. S2 Phonon dispersion of monolayer $NbOI_2$. ---------- 6

Fig. S3 Energy per cell against time step for a molecular dynamics calculation performed on $NbOI_2$ at 298K. ---------- 7

Table S3 Details of structural parameters for DFT-optimized structures of monolayer $NbOX_2$. ---------- 8

Table S4 Piezoelectric stress tensor elements ($\boldsymbol{e}_{11}$) and piezoelectric strain tensor elements ($\boldsymbol{d}_{11}$) of bulk $NbOX_2$. ---------- 8

Table S5 Piezoelectric tensor elements and electromechanical coupling factor of bulk $NbOX_2$. ---------- 9

Table S6 Dielectric constants of monolayer and bulk $NbOX_2$, computed by DFT. ---------- 9

Table S7 Stiffness Tensor elements C_{11}, C_{12}, C_{22} of monolayer $NbOX_2$ in the unit of $N\ m^{-1}$ and Compliance tensor elements S_{11}, S_{12}, S_{22}, S_{66} in $m\ N^{-1}$. ---------- 10

Table S8 Summary of piezoelectric coefficients from experiments. ---------- 10

Table S9 Comparison between 2D and 3D piezoelectric strain moduli $|d_{ij}|_{max}$ of 2D materials and their corresponding bulk piezoelectric parents. ---- 11

Fig. S4 Correlation between 2D and 3D piezoelectric stress moduli $|e_{ij}|_{max}$ of 2D materials and their corresponding bulk piezoelectric parents. ---- 11

Table S10 Numerical values of 2D and 3D piezoelectric stress moduli $|e_{ij}|_{max}$ of 2D materials and their corresponding bulk piezoelectric parents, as presented in Fig. S4. ---------- 12

Table S11 Details of structural parameters for bulk $NbOX_2$. ---------- 13

Fig. S5 Optical images of exfoliated $NbOX_2$ nanosheets. ---------- 13

Fig. S6 PFM measurements on 82-nm-thick $NbOI_2$. ---------- 14

Fig. S7 PFM measurements on 23-nm-thick $NbOI_2$. ---------- 14

Fig. S8 PFM measurements on 98-nm-thick $NbOI_2$. ---------- 15

Fig. S9 PFM measurements on 4.3-nm-thick $NbOI_2$. ---------- 15

Fig. S10 PFM measurements on 72-nm-thick $NbOCl_2$. ---------- 16

Fig. S11 Vector PFM Measurements on 17.2-nm-thick $NbOCl_2$. ---------- 16

Fig. S12 AFM measurements on $NbOX_2$. ---------- 17

Fig. S13 Measurement of the piezoelectric coefficients of α-In_2Se_3 and $CuInP_2S_6$ (CIPS) using a laser scanning vibrometer (LSV). ---------- 17

Fig. S14 Comparison between theoretical (DFT) and experimental maximal piezoelectric strain tensor elements ($\boldsymbol{d}_{ij}$). ---------- 18

Table S12 Electronic and ionic contributions to $\boldsymbol{e}_{11}$ of $NbOX_2$. ---------- 18

Fig. S15 Static charges on Nb and O atoms in $NbOI_2$ and $NbOCl_2$, plotted as a function of δx. ---------- 19
Fig. S16 Integrated crystal orbital overlap population (ICOOP) for the Nb-O bond in $NbOX_2$. ---------- 20
Table S13 Dynamical charge ($Z^*_{m^x,1}$) and $\frac{\partial u_{m^x}}{\partial \eta_1}$ of each atom in $NbOX_2$. ---------- 21
Fig. S17 High-throughput calculation results for maximum out-of-plane sheet piezoelectric tensor elements. ---------- 21
Table S14 Materials with large $\boldsymbol{e}_{3j}$. ---------- 22
Supplementary Discussions ---------- 22
Formalisms ---------- 22
Supplementary Note 1: Polarization switching in $NbOX_2$ ---------- 24
Fig. S18 Polarization versus electric field (P–E) loops of bulk $NbOI_2$ sheets at room temperature. ---------- 25
Fig. S19 Spectroscopic in-plane PFM switching loops of $NbOX_2$. ---------- 26
Supplementary Note 2: Ferroelectric-paraelectric phase transition in $NbOI_2$ - 26
Fig. S20 Temperature-dependent properties of $NbOI_2$. ---------- 27
Supplementary Note 3: Pseudo-Jahn-Teller Effect ---------- 28
Fig. S21 Valence and conduction band pairs involved in inducing the symmetry-breaking distortion though the PJTE. ---------- 28
Supplementary Note 4: Strain-assisted Ferroelectric Switching ---------- 29
Fig. S22 Effect of strain on the Nb-O bond lengths in $NbOX_2$. ---------- 29
High Throughput Calculation Results ---------- 30
Table S15 Table of quantities obtained from high throughput calculation workflow. ---------- 30
References ---------- 33

I. Supplementary Tables and Figures

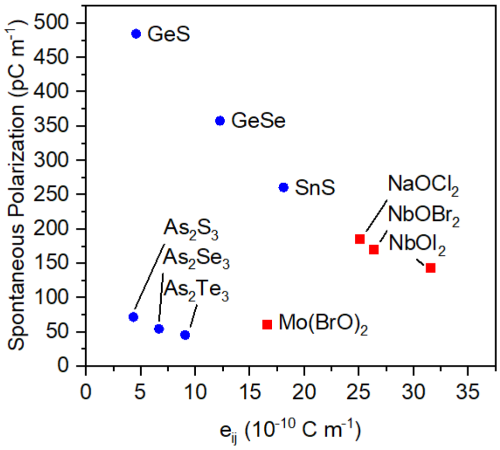

Fig. S1 | Plot of spontaneous polarization ($pC\ m^{-1}$) and maximum sheet e_{ij} for various 2D materials. Red squares denote data obtained from this study, blue dots denote data obtained from references[1, 2, 3].

Table S1 | Values of relaxed-ion e_{ij} ($10^{-10}\ C\ m^{-1}$) under different definitions. RI1 and RI2 are different definitions of relaxed-ion schemes. In RI1, the e_{ij} is obtained using the Berry Phase method by straining one axis, allowing the ionic positions to relax, while not allowing the unstrained lattice vectors to relax. In RI2, the unstrained lattice is allowed to relax to account for the Poisson effect. The values of e_{ij} obtained in these schemes are compared to those obtained through DFPT and by Fei et al. We note that the Poisson effect reduces the effective e_{22} for GeSe significantly.

Material	DFPT	RI1	RI2	Ref
GeSe	12.4	12.1	~0.3	12.3[3]
SnSe	28.2	28.6	14.1	34.9[3]
$NbOCl_2$	25.4	25.8	23.3	
$NbOBr_2$	26.4	27.3	27.1	
$NbOI_2$	31.6	33.3	31.4	

Table S2 | Relaxed-ion piezoelectric tensor element (e_{ij}), piezoelectric strain tensor element (d_{ij}), and spontaneous polarization (P_i) of selected materials in units of $10^{-10}\ C\ m^{-1}$, $pm\ V^{-1}$ and $pC\ m^{-1}$ respectively. In the cases where lattice orientations used in this study is different from existing literature, indices of tensor elements from this study are used.

		e_{ij}		d_{ij}		P_i
Material	ij	This study	Other studies	This Study	Other studies	
1H-MoS_2	11	3.72	3.64[4]		3.73[4]	
1H-$MoSe_2$	11	3.84	3.92[4]		4.72[4]	
1H-WS_2	11	2.54	2.47[4]		2.19[4]	
1H-WSe_2	11	2.60	2.71[4]		2.79[4]	
1H-BN	11	1.46	1.38[4]		0.60[4]	
InSe	22	0.85	0.57[5]		1.46[5]	
GaS	22	1.87	1.34[5]		2.06[5]	
GaSe	22	1.79	1.47[5]		2.30[5]	
$NbOCl_2$	11	25.1		27.4		185
$NbOBr_2$	11	26.4		30.0		170
$NbOI_2$	11	31.6		42.2		143
GeS	11		4.6[3]		75.43[3]	484[2]
GeSe	11	12.3	12.3[3]		212[3]	357[2]
SnS	11		18.1[3]		144.76[3]	260[2]
As_2S_3	22	1.72	4.36[1]		55.7[1]	71[1]
As_2Se_3	22	3.15	6.71[1]		61.7[1]	54[1]
As_2Te_3			9.09[1]		61.9[1]	21.6[1]
Bulk CIPS (0K DFT)	33		1.85[6]		18[6]	
Bulk CIPS (Exp)	33		36.7[6]		110[6]	

Here we note the disparity in the e_{22} values for As_2X_3 (X=S, Se) obtained in our study and those by Gao et al.[1] and attribute the disparity to the use of different exchange-correlation functionals.

In the figures below, we verify the dynamical stability of $NbOI_2$ by making sure there are no imaginary phonon frequencies for all **q** in the phonon dispersion spectra as well as by ensuring that there is no bond breaking in the molecular dynamics calculation.

The phonon dispersion of $NbOI_2$ is calculated using Quantum Espresso[7, 8, 9] working with a Projector Augmented Wave (PAW) approach[10] with the Perdew, Becke and Ernzerhof (PBE) Generalized Gradient Approximation (GGA) of the exchange-correlation functional[11]. We use a kinetic energy cut-off of 60 Ry for the plane wave basis set and a Monkhorst-Pack k point meshes of 12×6×1 is used. The atomic coordinates are fully relaxed using the conjugate gradient scheme until the maximum energy difference between iterations is less than 10^{-14} Ry and the residual force is less than 0.0001 Ry/Bohr.

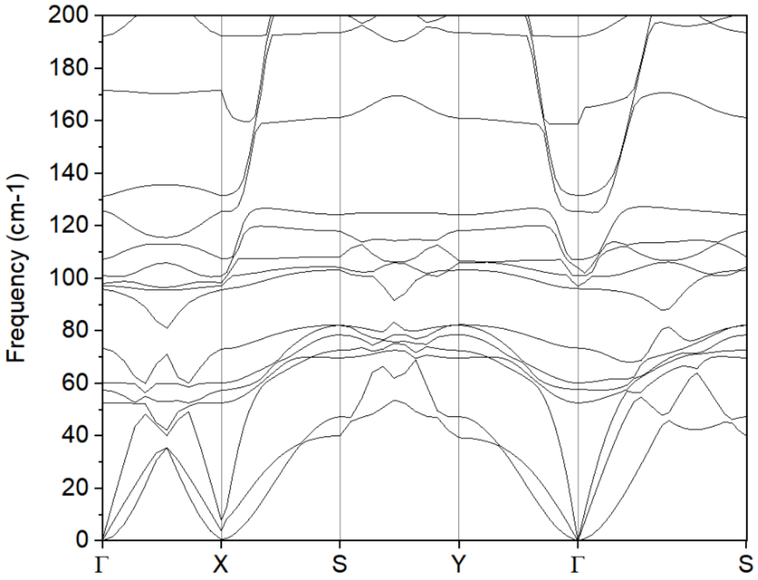

Fig. S2 | Phonon dispersion of monolayer $NbOI_2$. No imaginary frequency is observed, hence confirming that the structure is dynamically stable.

Ab initio molecular dynamics of $NbOI_2$ is performed using the VASP code[12] using the same set up as the targeted study on $NbOX_2$. Here, we use a 4×2×1 supercell and a Monkhorst-Pack k point mesh[13] with a density of 3×3×1. We use the NVT ensemble at 298 K with the Nose-Hoover thermostat for 5ps and a timestep of 1fs.

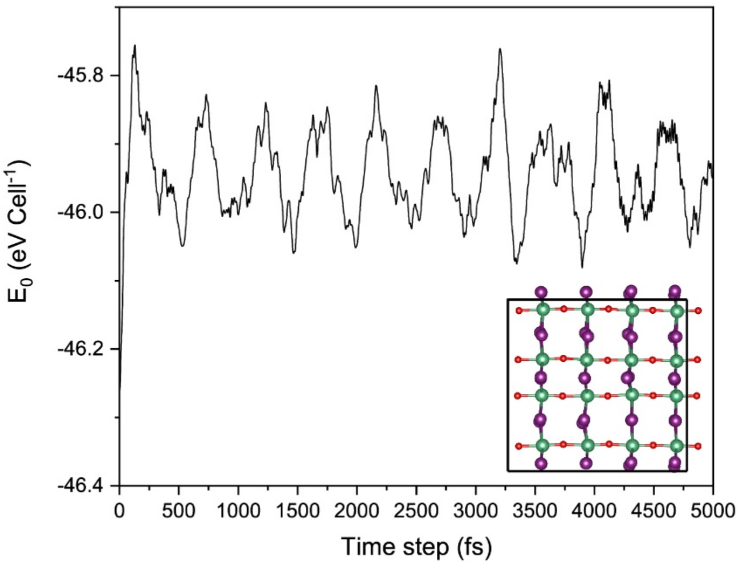

Fig. S3 | Energy per cell against time step for a molecular dynamics calculation performed on $NbOI_2$ at 298K. A 4 × 2 supercell is used for the simulation. The total energy of the system fluctuates about a fixed value, suggesting a dynamically stable structure. The inset shows the final structure after 5000 time steps of 1fs each, revealing no broken bonds, hence suggesting a dynamically stable structure at 298K.

Table S3 | Details of structural parameters for DFT-optimized structures of monolayer $NbOX_2$. ***a*** and ***b*** denote the in-plane lattice vectors in the polar (*x)* and non-polar (*y)* directions respectively; l_1 and l_2 are, respectively, the long and short Nb-O bond lengths in Figure 3. The magnitude of δ_x^{eqm} decreases down the halogen group with $NbOCl_2$ exhibiting the largest Nb displacement from the high symmetry point where $\delta x = 0$ Å and $NbOI_2$ exhibiting the least.

	$NbOCl_2$	$NbOBr_2$	$NbOI_2$
$\boldsymbol{a}$ (Å)	3.964	3.964	3.973
$\boldsymbol{b}$ (Å)	6.677	7.096	7.595
l_1 (Å)	2.142	2.133	2.123
l_2 (Å)	1.824	1.831	1.850
δ_x^{eqm} (Å)	0.318	0.302	0.274

Table S4 | Piezoelectric stress tensor elements ($\boldsymbol{e}_{11}$) and piezoelectric strain tensor elements ($\boldsymbol{d}_{11}$) of bulk $NbOX_2$. $\boldsymbol{e}_{11}$ of the bulk $NbOX_2$ is presented in both bulk units ($C\ m^{-2}$) as well as sheet units ($10^{-10}\ C\ m^{-1}$), where for comparison with $\boldsymbol{e}_{11}$ in the monolayer, we multiply the bulk $\boldsymbol{e}_{11}$ by half of the cell height to obtain an equivalent sheet value.

	$\boldsymbol{e}_{11}$ $(C\ m^{-2})$	sheet $\boldsymbol{e}_{11}$ $(10^{-10}\ C\ m^{-1})$	$\boldsymbol{d}_{11}$ $(pC\ N^{-1})$
$NbOCl_2$	3.56	25.3	27.4
$NbOBr_2$	3.46	26.6	30.1
$NbOI_2$	3.76	31.3	42.0

Table S5 | Piezoelectric tensor elements and electromechanical coupling factor of bulk $NbOX_2$. Here we report values obtained from two approximations (PBE and PBE-D3) to the exchange-correlation functional.

	PBE			PBE-D3		
	$NbOCl_2$	$NbOBr_2$	$NbOI_2$	$NbOCl_2$	$NbOBr_2$	$NbOI_2$
e_{11} $(C\,m^{-2})$	3.6	3.4	3.8	4.5	4.6	5.3
e_{12} $(C\,m^{-2})$	-0.1	-0.1	-0.1	-0.2	-0.2	-0.2
e_{13} $(C\,m^{-2})$	-0.1	0.0	0.0	-0.1	-0.1	-0.1
e_{26} $(C\,m^{-2})$	0.1	0.1	0.1	0.1	0.1	0.1
e_{35} $(C\,m^{-2})$	0.0	0.0	0.0	0.0	0.0	0.0
d_{11} $(pm\,V^{-1})$	27.3	30.1	41.8	27.3	30.2	43.6
d_{12} $(pm\,V^{-1})$	-4.0	-3.7	-4.7	-4.2	-4.2	-5.5
d_{13} $(pm\,V^{-1})$	-16.9	-21.0	-21.7	-11.0	-10.6	-13.2
d_{26} $(pm\,V^{-1})$	5.9	6.1	5.4	5.6	5.9	5.0
d_{35} $(pm\,V^{-1})$	4.1	4.9	4.0	1.2	1.5	1.5
k	0.95	0.96	1.07	0.93	0.92	1.02

Table S6 | Dielectric constants of monolayer and bulk $NbOX_2$, computed by DFT. Values for monolayer are caluclated with PBE and is scaled with respect to the thickness of the monolayer while values for bulk are calculated with both PBE and PBE-D3.

	Monolayer (PBE)			Bulk (PBE)			Bulk (PBE-D3)		
	$NbOCl_2$	$NbOBr_2$	$NbOI_2$	$NbOCl_2$	$NbOBr_2$	$NbOI_2$	$NbOCl_2$	$NbOBr_2$	$NbOI_2$
ε_{xx}	11.9	12.5	15.7	12.1	12.6	15.5	16.2	18.4	25.0
ε_{yy}	11.1	11.1	11.9	11.2	11.1	12.0	13.5	13.8	14.4
ε_{zz}	1.9	1.9	2.0	3.2	3.5	4.2	4.3	5.1	6.3

Table S7 | Stiffness Tensor elements C_{11}, C_{12}, C_{22} of monolayer $NbOX_2$ in the unit of N m^{-1} and Compliance tensor elements S_{11}, S_{12}, S_{22}, S_{66} in m N^{-1}.

	C_{11}	C_{12}	C_{22}	C_{66}	S_{11}	S_{12}	S_{22}	S_{66}
$NbOCl_2$	92.9	5.3	62.0	15.2	10.8	-1.1	15.5	65.9
$NbOBr_2$	89.0	5.6	63.1	14.4	11.3	-1.0	15.9	69.2
$NbOI_2$	75.6	5.3	62.0	13.6	13.3	-1.1	16.2	73.5

It can be observed that the Young's Modulus along the $\boldsymbol{a}_x$ axis (i.e. C_{11}) decreases by about 19% down the halogen group from 92.9 N m^{-1} for $NbOCl_2$ to 75.6 N m^{-1} for $NbOI_2$, while that along $\boldsymbol{a}_y$ axis (C_{22}) does not change significantly.

Table S8 | Summary of piezoelectric coefficients from experiments. The precision of the magnitudes are kept at the levels reported in the references.

Material	Piezoelectric coefficient	Magnitude	Reference
h-MoS_2	$\boldsymbol{e}_{11}$	2.9×10^{-10} C m^{-1}	14
h-BN	$\boldsymbol{e}_{11}$	2.91×10^{-10} C m^{-1}	15
h-MoSSe	$\boldsymbol{d}_{33}$	0.1 pm V^{-1}	16
WTe_2	$\boldsymbol{d}_{33}$	0.7 pm V^{-1}	17
g-C_3N_4	$\boldsymbol{d}_{33}$	1 pm V^{-1}	18
CdS	$\boldsymbol{d}_{33}$	16.4 pm V^{-1}	19
SnSe	$\boldsymbol{d}_{11}$	23 pm V^{-1}	20
ZnO nanobelt	$\boldsymbol{d}_{33}$	14.3 – 26.7 pm V^{-1}	21
α-In_2Se_3	$\boldsymbol{d}_{33}$	5.3 pm V^{-1}	This Work
$CuInP_2S_6$	$\boldsymbol{d}_{33}$	4.1 pm V^{-1}	This Work
$NbOCl_2$	$\boldsymbol{d}_{11}$	9.4 pm V^{-1}	This Work
$NbOI_2$	$\boldsymbol{d}_{11}$	21.45 pm V^{-1}	This Work

Table S9 | Comparison between 2D and 3D piezoelectric strain moduli $|\boldsymbol{d_{ij}}|_{max}$ of 2D materials and their corresponding bulk piezoelectric parents. Values for 2D $|\boldsymbol{d}_{ij}|_{max}$ are obtained as from **Fig. 3a**. Values of 3D $|\boldsymbol{d}_{ij}|_{max}$ for InSe and GaSe are obtained from Materials Project; Values of 3D $|\boldsymbol{d}_{ij}|_{max}$ for $NbOX_2$ are obtained from $\boldsymbol{d}_{11_{PBE-D3}}$ of **Table S5**. The materials project material ID of the parent materials are presented in square brackets.

Material	2D $|\boldsymbol{d}_{ij}|_{max} (pm\ V^{-1})$	3D $|\boldsymbol{d}_{ij}|_{max}\ (pm\ V^{-1})$
InSe	1.5	2.3 [mp-22691]
GaSe	2.3	2.9 [mp-11342]<br>3.3 [mp-1572]
$NbOCl_2$	27.4	27.3 [mp-549720]
$NbOBr_2$	30.0	30.2 [mp-550070]
$NbOI_2$	42.2	43.6 [mp-1025567]

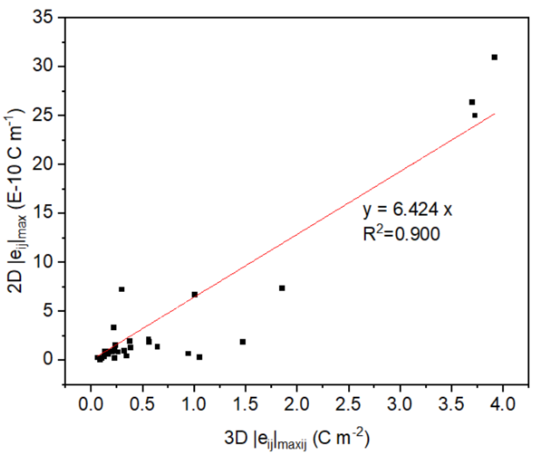

Fig. S4 | Correlation between 2D and 3D piezoelectric stress moduli $|\boldsymbol{e_{ij}}|_{max}$ of 2D materials and their corresponding bulk piezoelectric parents. The linear trend line passing through the origin has a gradient of ~6.4Å. Numerical details of each datapoint can be obtained from **Table S10**. Unlike the piezoelectric strain coefficients, the piezoelectric stress coefficients are defined differently for 2D and 3D systems, as described in the Formalisms section. The gradient of ~6.4Å corresponds to the average thickness of a monolayer.

Table S10 | Numerical values of 2D and 3D piezoelectric stress moduli $|e_{ij}|_{max}$ of 2D materials and their corresponding bulk piezoelectric parents, as presented in Fig. S4. Headings of the first 3 columns are the exact database dictionary keys used in this database. Heading of the last column is in the format MP_{dictionary_key_in_materials_project}.

formula_pretty	*mat_project_id*	*dielectric.max_abs_sheet_piezo* ($E\text{-}10\ C\ m^{-1}$)	*MP_piezo.eij_max* ($C\ m^{-2}$)
Li_2WS_4	mp-753195	0.0583	0.0902
Cu_2WSe_4	mp-1025340	0.2071	0.2319
$LiBH_4$	mp-644223	0.2221	0.1023
Cu_2WS_4	mp-8976	0.2314	0.0621
$NaHO$	mp-626000	0.2808	1.0514
$Sn(PS_3)_2$	mp-36381	0.3645	0.1243
H_3BrO	mp-625521	0.3860	0.3468
$ZrCl_2$	mp-23162	0.6390	0.1678
$CaHClO$	mp-642725	0.6500	0.9465
$BiTeCl$	mp-28944	0.8225	0.2654
$InSe$	mp-22691	0.8475	0.1331
Ta_3TeI_7	mp-29117	0.8632	0.2082
Nb_3SBr_7	mp-29057	0.8645	0.1626
Hg_3AsSe_4Br	mp-567949	0.9225	0.3205
Nb_3TeI_7	mp-29689	0.9446	0.2286
$BiTeBr$	mp-33723	1.2412	0.3846
$Hg_2P_2S_7$	mp-27171	1.3647	0.6476
$B_2S_2O_9$	mp-1019509	1.5431	0.2371
$ZrGeTe_4$	mp-13542	1.8207	0.5666
$AlHO_2$	mp-625054	1.8717	1.4761
$BiTeI$	mp-22965	1.9007	0.3763
$HfGeTe_4$	mp-567817	2.1278	0.5587
$InGaS_3$	mp-19885	3.3485	0.2260
$NbTlBr_4O$	mp-551826	6.7080	1.0059
Sn_2IF_3	mp-27167	7.2032	0.3021
$InSnCl_3$	mp-998560	7.3495	1.8560
$NbCl_2O$	mp-1025567	24.9742	3.7281
$NbBr_2O$	mp-550070	26.3286	3.7007
NbI_2O	mp-549720	30.9221	3.9211
SbF_3	mp-1880	46.0720	1.5282

Table S11 | Details of structural parameters for bulk $NbOX_2$. DFT values are compared with those deduced from single-crystal X-ray diffraction in this work and in the ICSD database[22, 23]. **a** and ***b*** denote the in-plane lattice vectors in the polar (x) and non-polar (y) directions respectively, ***c*** denotes the out-of-plane lattice vector; α, β and γ denote the angles ∠**bc,** ∠**ac** and ∠**ab** respectively. l_1 and l_2 are, respectively, the long and short Nb-O bond lengths. The magnitude of δ_x^{eqm} decreases down the halogen group with $NbOCl_2$ exhibiting the largest Nb displacement from the high symmetry point where $\delta x = 0$ Å and $NbOI_2$ exhibiting the least.

	DFT (PBE)			DFT (PBE-D3)			Experiment		ICSD	
	$NbOCl_2$	$NbOBr_2$	$NbOI_2$	$NbOCl_2$	$NbOBr_2$	$NbOI_2$	$NbOCl_2$	$NbOI_2$	$NbOBr_2$	$NbOI_2$
**a** (Å)	3.963	3.963	3.974	3.939	3.932	3.943	3.904	3.933	3.908	3.924
**b** (Å)	6.773	7.096	7.601	6.744	7.065	7.558	6.720	7.523	7.023	7.520
**c** (Å)	15.180	16.397	17.669	13.568	14.553	15.914	12.863	15.188	13.833	15.184
α (°)	104.3	105.0	105.3	105.1	105.0	104.9	105.6	105.4	105.0	105.5
β (°)	105.1	104.0	103.0	90.0	90.0	90.0	90.0	90.0	90.0	90.0
γ (°)	90.0	90.0	90.0	90.0	90.0	90.0	90.0	90.0	90.0	90.0
l_1 (Å)	2.140	2.132	2.125	2.111	2.094	2.081	2.125	2.106	2.110	2.110
l_2 (Å)	1.824	1.831	1.850	1.829	1.840	1.863	1.779	1.827	1.800	1.810
δ_x^{eqm} (Å)	0.316	0.301	0.276	0.282	0.254	0.218	0.346	0.279	0.310	0.300

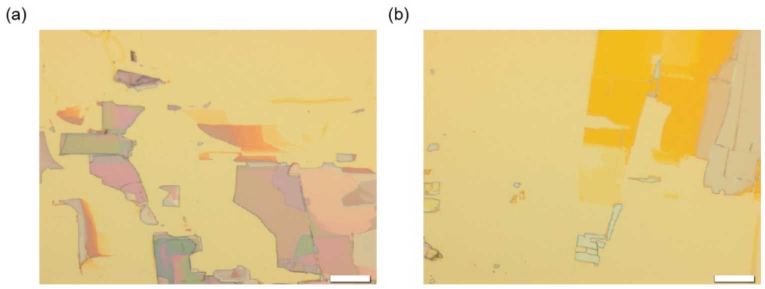

Fig. S5 | Optical images of exfoliated $NbOX_2$ nanosheets. **(a)** $NbOI_2$ flakes on Au substrate. **(b)** $NbOCl_2$ flakes on Au substrate.

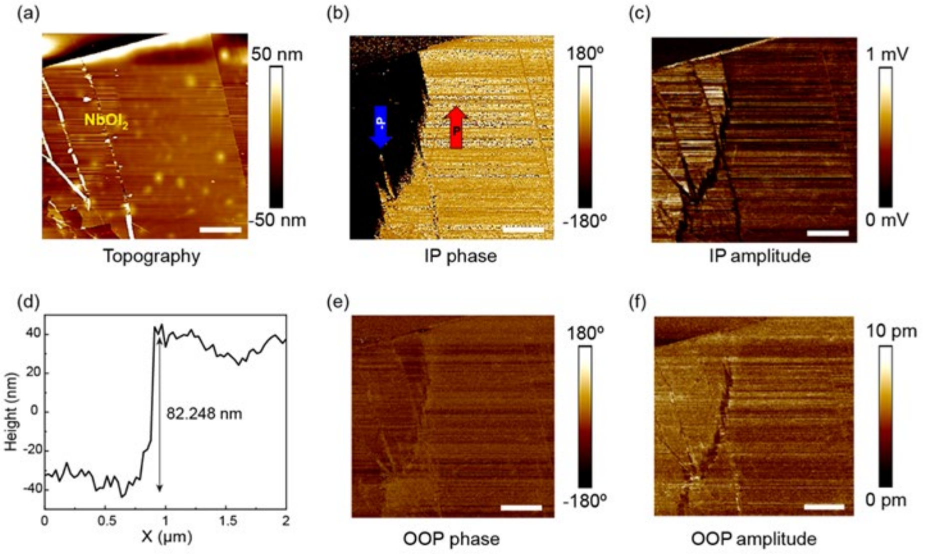

Fig. S6 | PFM measurements on 82-nm-thick $NbOI_2$. Topography, height profile, in-plane (IP) phase, IP amplitude, out-of-plane (OOP) phase, and OOP amplitude of 82-nm-thick $NbOI_2$ flake. Scale bars: 4 μm. Drive voltage: 5 V. Drive frequency: 65 kHz.

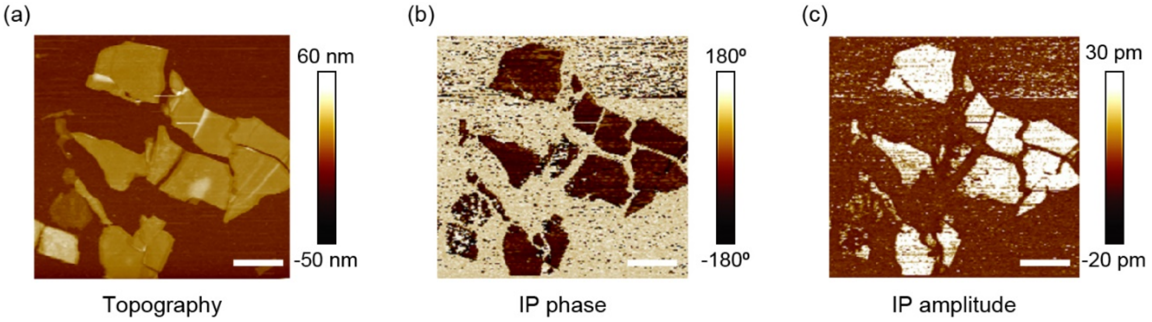

Fig. S7 | PFM measurements on 23-nm-thick $NbOI_2$. Topography, in-plane (IP) phase, and IP amplitude images of 23-nm-thick $NbOI_2$ flake. Scale bars: 4 μm. Drive voltage: 2.5 V. Drive frequency: 866 kHz.

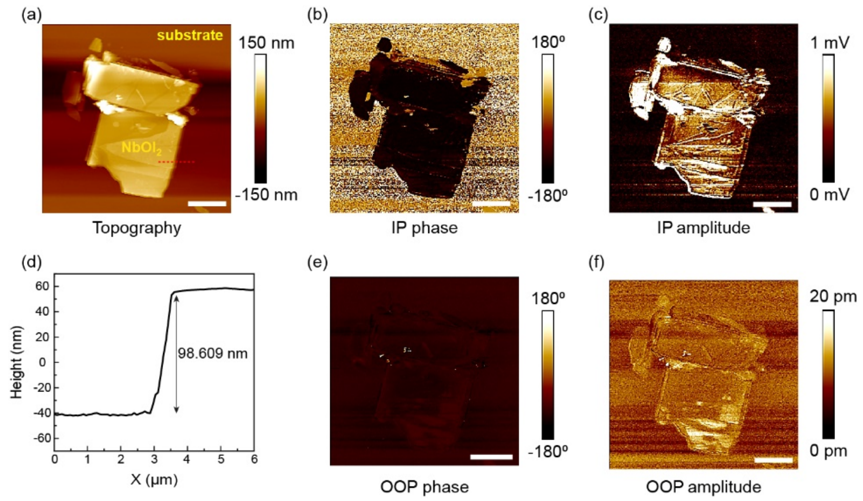

Fig. S8 | PFM measurements on 98-nm-thick $NbOI_2$. Topography, height profile, in-plane (IP) phase, IP amplitude, out-of-plane (OOP) phase, and OOP amplitude of 98-nm-thick $NbOI_2$ flake. Scale bars: 4 μm. Drive voltage: 10 V. Drive frequency: 75 kHz.

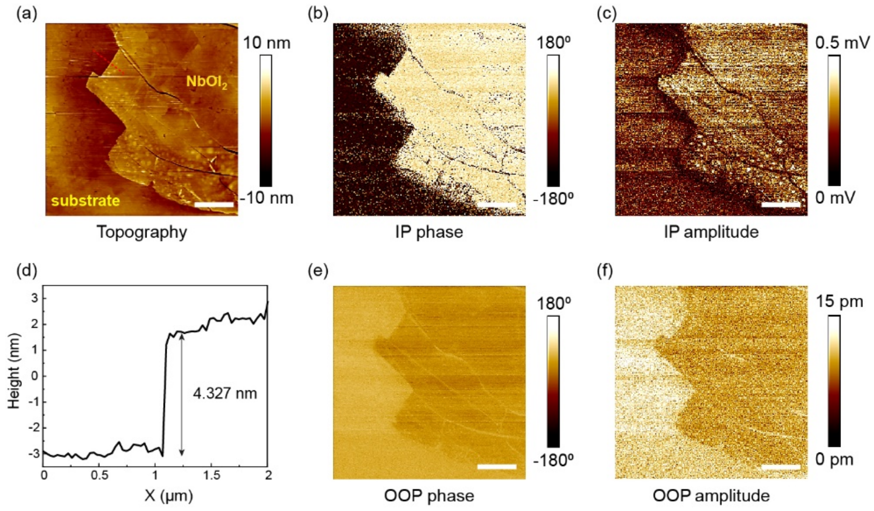

Fig. S9 | PFM measurements on 4.3-nm-thick $NbOI_2$. Topography, height profile, in-plane (IP) phase, IP amplitude, out-of-plane (OOP) phase, and OOP amplitude of 4.3-nm-thick $NbOI_2$ flake. Scale bars: 2 μm. Drive voltage: 10 V. Drive frequency: 30.5 kHz.

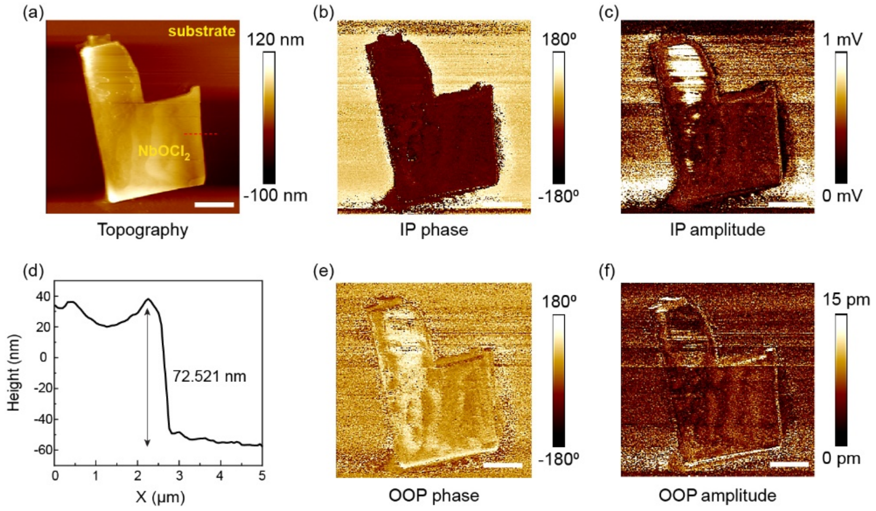

Fig. S10 | PFM measurements on 72-nm-thick $NbOCl_2$. Topography, height profile, in-plane (IP) phase, IP amplitude, out-of-plane (OOP) phase, and OOP amplitude of 72-nm-thick $NbOCl_2$ flake. Scale bars: 3 μm. Drive voltage: 10 V. Drive frequency: 75 kHz.

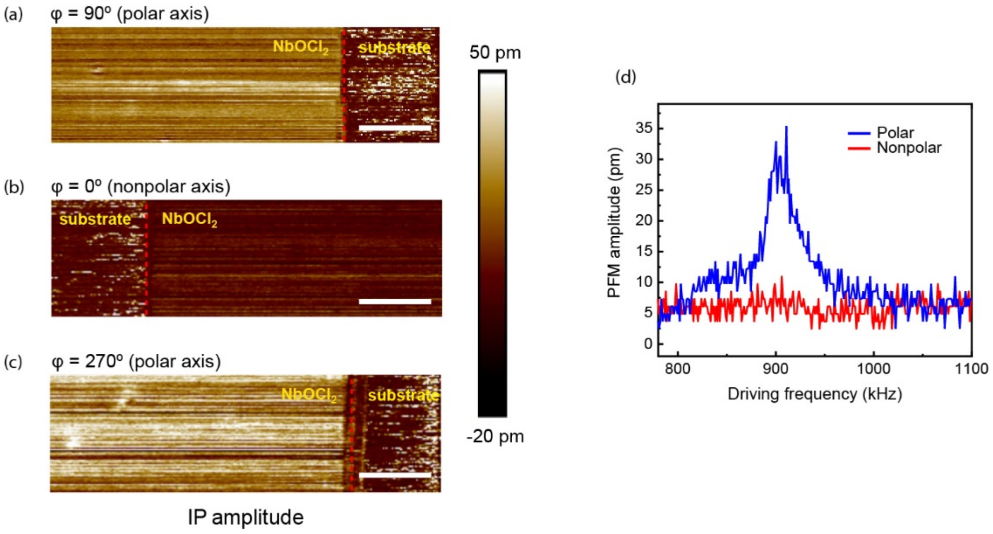

Fig. S11 | Vector PFM Measurements on 17.2-nm-thick $NbOCl_2$. (a-c) Vector PFM IP amplitude images of 17.2-nm-thick $NbOCl_2$ showing spontaneous polarization at 90° (a), 0° (b), and 270° (c) angles relative to the cantilever long axis. **(d)** PFM amplitude profiles along the polar and nonpolar axes of the $NbOCl_2$ flake. Scale bars are 2 μm.

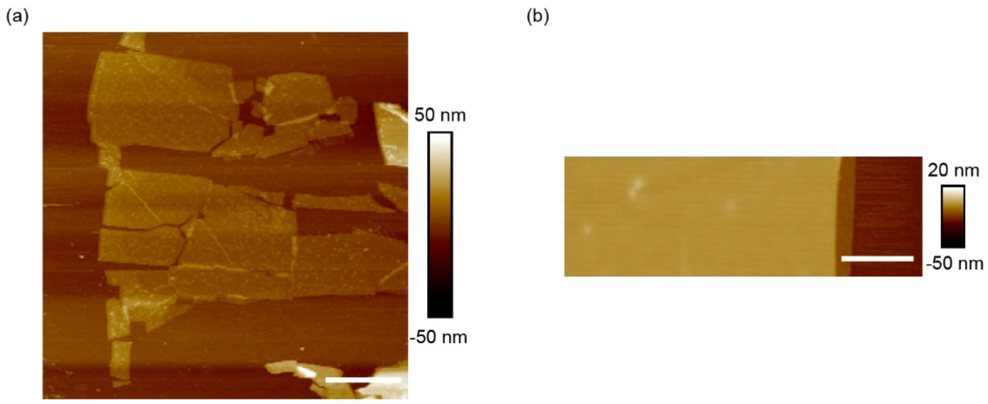

Fig. S12 | AFM measurements on $NbOX_2$. Topography images of **(a)** the 10-nm-thick $NbOI_2$ flakes shown in **Fig. 4**e-g and **(b)** the 17.2-nm-thick $NbOCl_2$ flake shown in **Fig. S11**. Scale bars: (a) 4 μm, (b) 2 μm.

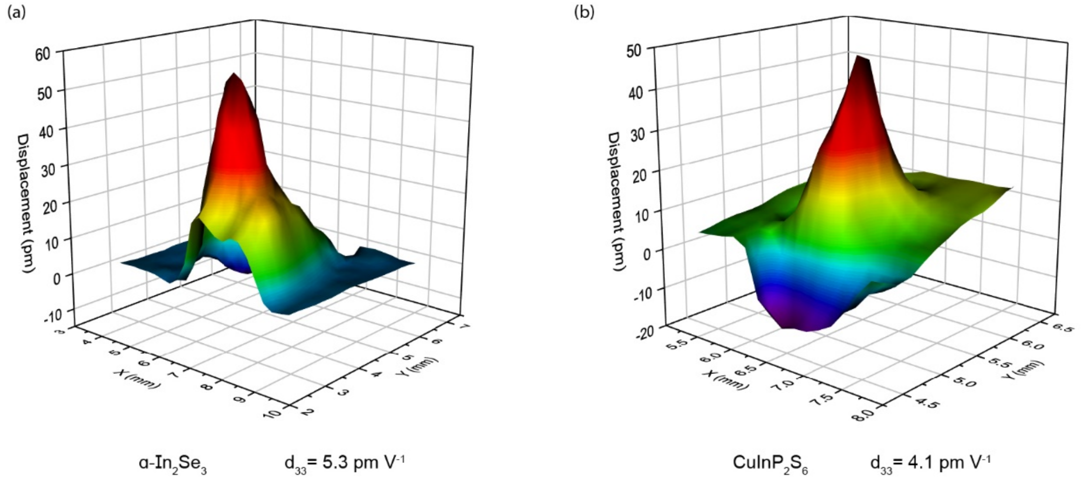

Fig. S13 | Measurement of the piezoelectric coefficients of α-In_2Se_3 and $CuInP_2S_6$ (CIPS) using a laser scanning vibrometer (LSV). 3D graphs of the instantaneous vibration when the displacement magnitude reaches the maximum under the sine-wave driving electrical signal. The measurements are conducted along the vertical polar directions d_{33} of α-In_2Se_3 **(a)** and CIPS **(b)**. The in-plane cofficients of α-In_2Se_3 are also measured and found to be $\boldsymbol{d}_{11}$ = 1.7 pm/V and $\boldsymbol{d}_{22}$ = 6.0 pm/V.

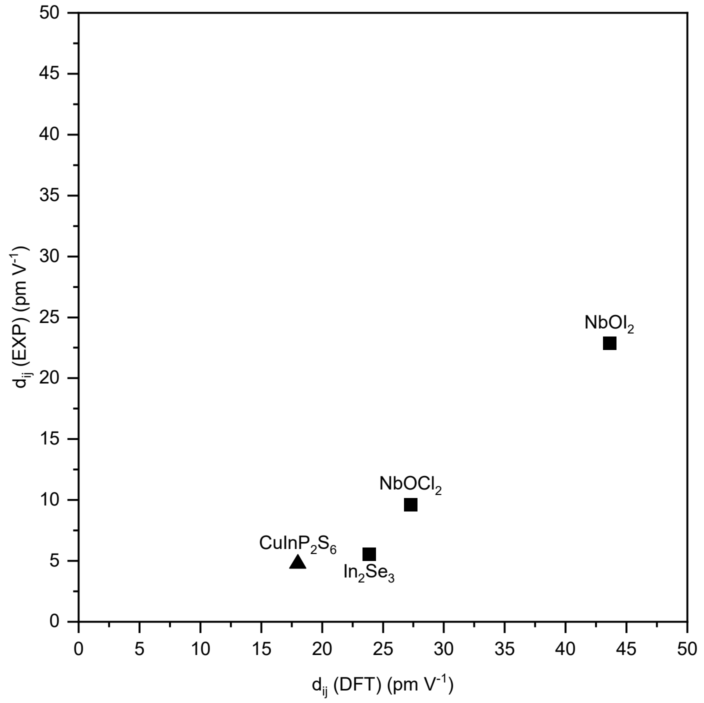

Fig. S14 | Comparison between theoretical (DFT) and experimental maximal piezoelectric strain tensor elements (d_{ij}). DFT value for d_{33} of $CuInP_2S_6$ is taken from ref [6]. Values for the other materials are obtained in this work. The horizontal and vertical axes of this figure are plotted in scale.

Table S12 | Electronic and ionic contributions to e_{11} of $NbOX_2$. The electronic contribution (e) refers to value of e_{11} computed from the "clamped ion" configuration.

e_{11}	e	ion	total
$NbOCl_2$	-0.4	25.5	25.1
$NbOBr_2$	-0.4	26.8	26.4
$NbOI_2$	-0.2	31.8	31.6

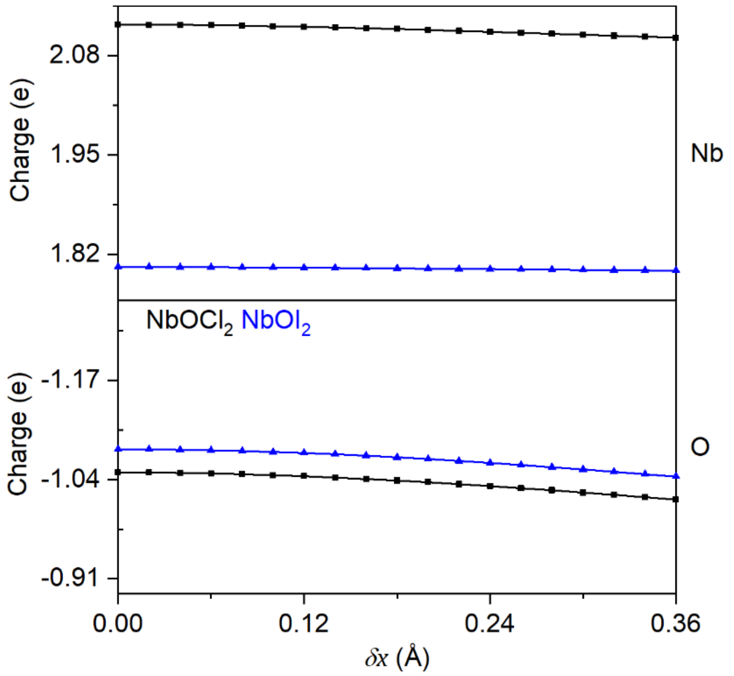

Fig. S15 | Static charges on Nb and O atoms in $NbOI_2$ and $NbOCl_2$, plotted as a function of δx. These charges are computed used the Bader approach[24, 25, 26, 27].

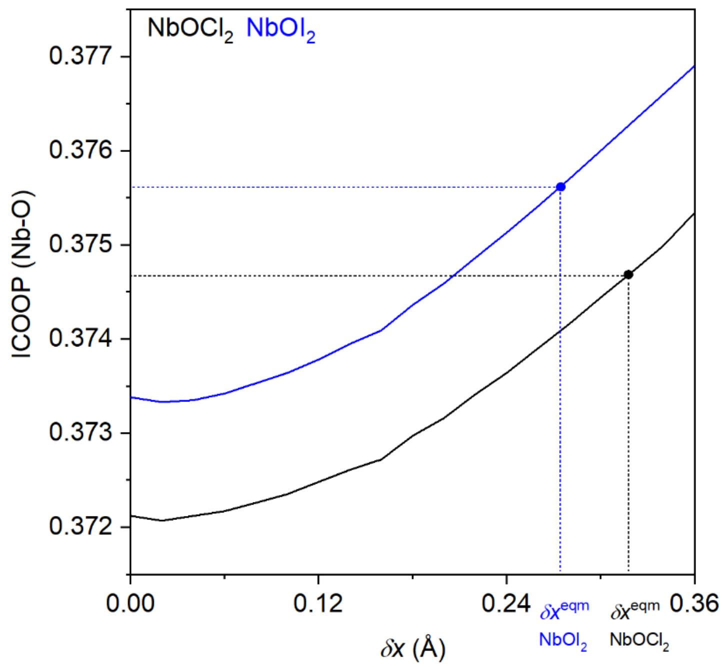

Fig. S16 | Integrated crystal orbital overlap population (ICOOP) for the Nb-O bond in $NbOX_2$. ICOOP is a measure of the degree of covalency in a bond, and a more positive value indicates greater covalency[28]. Increasing covalence in the Nb-O bond is observed as Nb is moved from the centred symmetric structure, with increasing δx. This observation is consistent with the pseudo-Jahn-Teller effect. At the same δx, Nb-O bonds are more covalent in $NbOI_2$ than in $NbOCl_2$, which can be explained by the larger electronegativity of Cl compared to I. Dotted lines denote the values for the equilibrium structures. Nb-O bonds in $NbOI_2$ are more covalent than those in $NbOCl_2$. The trend for $NbOBr_2$ falls between those of $NbOI_2$ and $NbOCl_2$ and is omitted here.

Table S13 | Dynamical charge ($Z^*_{m^x,1}$) and $\frac{\partial u_{m^x}}{\partial \eta_1}$ of each atom in NbOX₂. m^x refers to the x component of each atomic displacement. We see that the values of $Z^*_{m^x,1}$ and $\frac{\partial u_{m^x}}{\partial \eta_1}$ have the same sign, contributing to a large value in the sum for e^{ion}_{11}. Note that m^x refers to the x-component of each atomic displacement.

	$Z^*_{m^x,1}$ (e)			$\frac{\partial u_{m^x}}{\partial \eta_1}$ (Å)		
	$NbOCl_2$	$NbOBr_2$	$NbOI_2$	$NbOCl_2$	$NbOBr_2$	$NbOI_2$
Nb1	7.550	7.713	8.081	2.109	2.354	3.023
Nb2	7.550	7.713	8.081	2.109	2.354	3.023
X1	-0.484	-0.381	-0.218	-0.933	-1.081	-1.410
X2	-0.382	-0.274	-0.084	-0.429	-0.558	-0.844
X3	-0.382	-0.274	-0.084	-0.429	-0.558	-0.844
X4	-0.484	-0.381	-0.218	-0.933	-1.081	-1.410
O1	-6.683	-7.058	-7.779	-0.747	-0.714	-0.769
O2	-6.683	-7.058	-7.779	-0.747	-0.714	-0.769

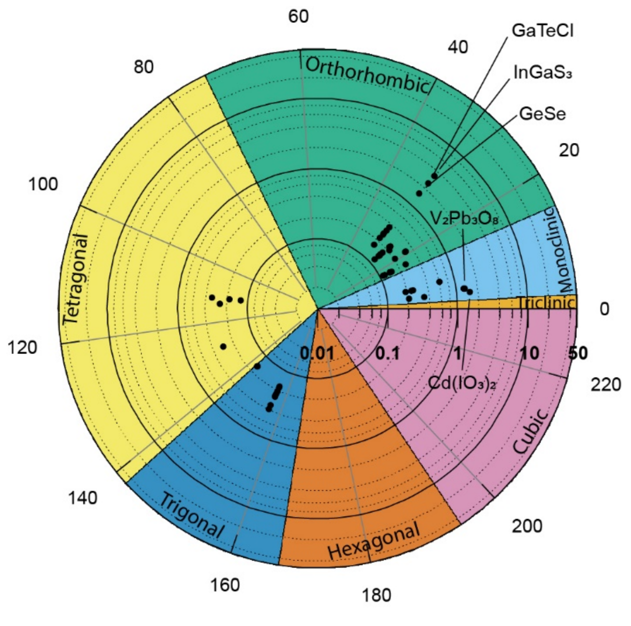

Fig. S17 | High-throughput calculation results for maximum out-of-plane sheet piezoelectric tensor elements. The radial axis represents the magnitude of e_{3j} in units of 10^{-10} C m^{-1} on a log scale and the angular axis represents the 230 space groups.

Table S14 | Materials with large e_{3j}.

Material	max sheet e_{3j} (10^{-10} C m^{-1})	
$InGaS_3$	3.349	e_{34}
GaTeCl	2.429	e_{34}
GeSe	1.557	e_{34}
$Cd(IO_3)_2$	1.546	e_{35}
$V_2Pb_3O_8$	1.330	e_{34}

II. Supplementary Discussions

Formalisms

In this section, $\boldsymbol{e}_{ij}$ refers to the three-dimensional (3D) relaxed-ion piezoelectric stress tensor, which is defined by the relation[29]

$$\boldsymbol{e}_{ij} = \left(\frac{\partial \boldsymbol{P}_i}{\partial \boldsymbol{\eta}_j}\right)\Bigg|_{\boldsymbol{\varepsilon}} = -\left(\frac{\partial \boldsymbol{\sigma}_i}{\partial \boldsymbol{\epsilon}_j}\right)\Bigg|_{\boldsymbol{\eta}} \qquad 1$$

where $\boldsymbol{P}$ is the electric polarization, $\boldsymbol{\eta}$ is the homogeneous strain, $\boldsymbol{\sigma}$ is the mechanical stress, and $\boldsymbol{\epsilon}$ is the homogeneous electric field. $i = \{x, y, z\}$) and $j = \{1 \dots 6\}$ as in Voigt notation.

$\boldsymbol{e}_{ij}$ consists of two parts: clamped-ion contributions ($\boldsymbol{e}_{ij}^{el}$) as well as ionic contributions ($\boldsymbol{e}_{ij}^{ion}$)[30].

$$\boldsymbol{e}_{ij} = \boldsymbol{e}_{ij}^{el} + \boldsymbol{e}_{ij}^{ion} \qquad 2$$

$\boldsymbol{e}_{ij}^{el}$ is a second-derivative response function tensor of energy (*E*) with respect to homogeneous electric field and homogeneous strain.

$$\boldsymbol{e}_{ij}^{el} = -\frac{\partial^2 E}{\partial \boldsymbol{\epsilon}_i \partial \boldsymbol{\eta}_j}\Bigg|_{u} \qquad 3$$

It is considered a clamped-ion quantity because the ionic coordinates ($\boldsymbol{u}$) are not relaxed when the homogeneous electric field and strain are applied, hence it represents the piezoelectric contributions from the electrons alone.

On the other hand, $\boldsymbol{e}_{ij}^{ion}$ is defined, in an implied sum notation, as

$$\boldsymbol{e}_{ij}^{ion} = \frac{1}{\Omega_0}\boldsymbol{Z}_{mi}^{*}\frac{\partial \boldsymbol{u}_m}{\partial \boldsymbol{\eta}_j} \qquad 4$$

where Ω_0 is the cell volume before deformation and $\boldsymbol{Z}_{mi}^{*}$ is the Born effective charge (m is a composite label for atom and displacement directions, ranging from 1 to 3N). $\boldsymbol{e}_{ij}^{ion}$ represents the ionic contribution to $\boldsymbol{e}_{ij}$ due to the relaxation of ionic positions after the application of strain.

$\boldsymbol{e}_{ij}$ is computed using density functional perturbation theory (DFPT)[30, 31, 32] where $\boldsymbol{e}_{ij}^{ion}$ is computed in terms of the pseudo inverse of force constant matrix ($\boldsymbol{K}_{mn}$) and the internal strain tensor ($\boldsymbol{\Lambda}_{nj}$) as shown in Equation S5) [30].

$$\boldsymbol{e}_{\boldsymbol{ij}}^{\boldsymbol{ion}} = \frac{1}{\Omega_0}\boldsymbol{Z}_{\boldsymbol{mi}}^{*}(\boldsymbol{K}^{-1})_{mn}\boldsymbol{\Lambda}_{\boldsymbol{nj}} \qquad 5$$

The piezoelectric strain tensor elements ($\boldsymbol{d}_{ij}$), frequently used in experimental studies, is defined by the relation[29]

$$\boldsymbol{d}_{ij} = \left(\frac{\partial \boldsymbol{P}_i}{\partial \boldsymbol{\sigma}_j}\right)\bigg|_{\boldsymbol{\varepsilon}} = -\left(\frac{\partial \boldsymbol{\eta}_i}{\partial \boldsymbol{\epsilon}_j}\right)\bigg|_{\sigma} \qquad 6$$

$\boldsymbol{d}_{ij}$ can be obtained from $\boldsymbol{e}_{ij}$ and elastic compliance tensor ($\boldsymbol{S}_{ij}$) through the following relationship

$$\boldsymbol{d}_{ij} = \boldsymbol{e}_{ik}\boldsymbol{S}_{kj} \qquad 7$$

The sheet $\boldsymbol{e}_{ij}$ defined in the main text is obtained by multiplying the 3D $\boldsymbol{e}_{ij}$ by the cell height.

The sheet $\boldsymbol{S}_{ij}$ defined in **Table S7** is obtained from 3D $\boldsymbol{S}_{ij}$ by setting elements related to the z-direction to 0 and dividing the rest of the elements by cell height[33].

Using both sheet $\boldsymbol{e}_{ij}$ and sheet $\boldsymbol{S}_{ij}$ for calculation of $\boldsymbol{d}_{ij}$ is equivalent to using their bulk counterparts because the scaling by height in both terms are cancelled off.

Note that when strain and electric field are simultaneously present, a more accurate formulation, as presented by Wu et al.[30], needs to be invoked. For the sake of readability, we present the "improper" formulation here while understanding that the "proper" terms as presented by Wu et al. are used in the DFPT calculations. Also, we note that the "improper" and "proper" terms are equivalent when $j = \{1, 2, 3\}$.

Supplementary Note 1: Polarization switching in $NbOX_2$

We study the polarization switching in bulk $NbOI_2$ microscopically via polarization *versus* electric field (P–E) measurements[34, 35] and locally through spectroscopic PFM characterizations. The P–E ferroelectric hysteresis loop of $NbOI_2$ when an external electric field is applied to its polar axis is displayed in **Fig. S18b**. When the applied electric field is in the vicinity of the coercive field E_c (~ 8.5 kV/cm), the polarization shows a drastic variation due to domain reversal, i.e., the field is large enough to switch domains with the unfavorable direction of polarization. Polarization reversal is a consequence of the motion of domain walls under the influence of strong applied fields. The process involves the redistribution of the volumes of energetically favorable and unfavorable domains. The ferroelectric domain wall motion and domain switching account for the hysteretic behavior of polarization. If the applied field strength slowly decreases, some domains would back-switch. At the zero-field point, the polarization is nonzero. The crystal reaches a zero-polarization state at the opposite E_c. Further increase of the field in the negative direction induces polarization switching in the opposite direction. The rounding of the

hysteresis loop is ascribed to the small bandgap of $NbOI_2$ and leakage-related issues. The P–E hysteresis disappears when the field is applied along the nonpolar axis of $NbOI_2$ (**Fig. S18c**).

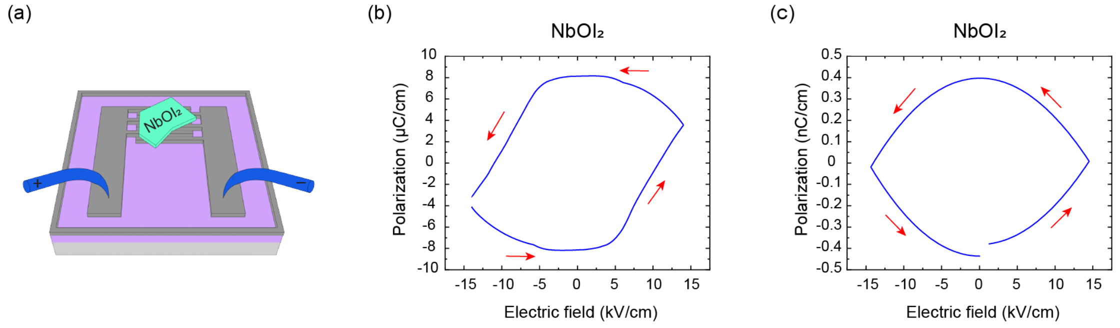

Fig. S18 | Polarization *versus* electric field (P–E) loops of bulk $NbOI_2$ sheets at room temperature. **(a)** Schematic illustration of P–E measurements on $NbOI_2$ lateral devices. The electrical field is applied along the in-plane polar **(b)** and nonpolar **(c)** axes of $NbOI_2$.

Scanning tip-induced switching events were recorded using spectroscopic PFM to further confirm the switching characteristics of the ultrathin $NbOX_2$ flakes. The in-plane hysteresis curves of $NbOI_2$ and $NbOCl_2$ are shown in **Fig. S19a** and **Fig. S19b**, respectively. The phase-electric field hysteresis loop elucidates the local polarization behavior while the amplitude-electric field hysteresis loop defines the local strain response. We find that the phase can be switched by 180 ° at + 2.5 V and switched back at - 3 V, and the amplitude response displays a butterfly-like hysteric loop with dips at the same voltages as the phase curve. Meanwhile, no obvious change in the surface morphology of the $NbOX_2$ nanoflakes is found during the field cycling. The pronounced in-plane polarization reversibility at the positive and negative coercive field points and the corresponding butterfly strain-electric field hysteresis, affirm the polarization switching in ultrathin $NbOX_2$.

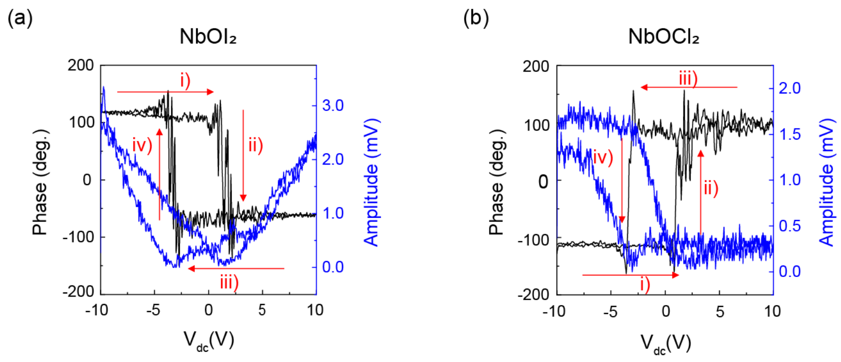

Fig. S19 | Spectroscopic in-plane PFM switching loops of $NbOX_2$. Spectroscopic in-plane PFM switching loops of exfoliated **(a)** 4.1-nm-thick $NbOI_2$ and **(b)** 7.8-nm-thick $NbOCl_2$.

Supplementary Note 2: Ferroelectric-paraelectric phase transition in $NbOI_2$

The ferroelectric-paraelectric phase transition in $NbOI_2$ was confirmed by temperature-dependent differential scanning calorimetry (DSC) and second harmonic generation (SHG) measurements. Differential scanning calorimetry (DSC) is a thermo-analytical technique that measures physical and chemical changes within a material in response to temperature. From the DSC heat flow curve, the changes in heat capacity that occurs around the ferroelectric-paraelectric phase transition can be identified as an exothermic or endothermic peak on the low-temperature side of the material's melting/sublimation peak. **Fig. S20a** depicts the DSC result of $NbOI_2$ during a heating cycle. An endothermic peak with a peak value of ~ 189.27 °C is observed in the DSC heat flow curve and assigned to the phase transition Curie temperature (T_c) of $NbOI_2$. This corresponds to a transition from ferroelectric phase with non-centrosymmetric C2 (No. 5) symmetry to paraelectric (PE) phase with centrosymmetric C2/m (No. 12) symmetry. SHG is highly sensitive to the inversion-symmetry breaking that accompanies a ferroelectric-paraelectric phase transition; only non-centrosymmetric structures are capable of emitting SHG light. We found that the SHG signal vanishes once T_c is exceeded and emerges again upon cooling (**Fig. S20b**).

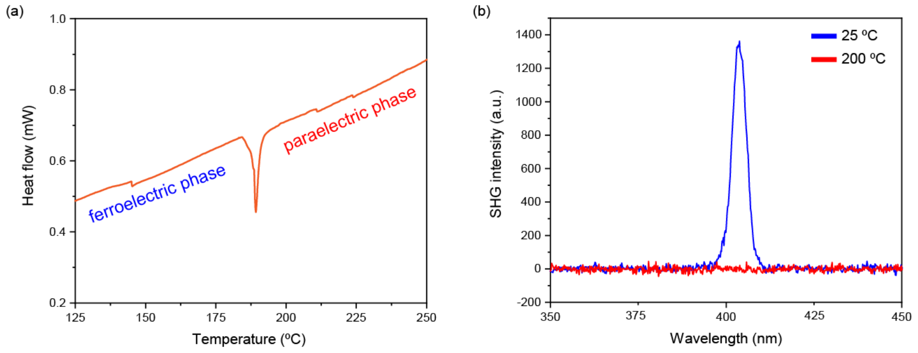

Fig. S20 | Temperature-dependent properties of $NbOI_2$. (a) DSC curve. **(b)** SHG spectra at 25 °C and 200 °C for the excitation wavelengths of λ_{pump} = 800 nm.

Supplementary Note 3: Pseudo-Jahn-Teller Effect

To understand the pseudo-Jahn-Teller effect (PJTE) driving the off-centre displacements of Nb in $NbOX_2$, we construct the centred symmetric structures, in which the atoms of $NbOX_2$ are relaxed with the Nb atoms constrained at $\delta x = 0$ Å, and study the valence and conduction band eigenstates. Comparing the orbital characters of these eigenstates with those of the equilibrium structures, we identify pairs of valence and conduction band states in the equilibrium structure, that are linear combinations of pairs of valence and conduction band states in the symmetric structure. This mixing of valence and conduction band states leads to an increased covalency as well as increased energy difference within each pair of states, and is accompanied by a spontaneous symmetry-breaking distortion[36] as observed here. The band indices of these valence and conduction band pairs (VB1, CB1 and VB2, CB2) are provided in **Fig. S21** below. These bands mainly comprise O p_y, Nb d_{xy}, O p_z and Nb d_{xz} orbitals respectively. In both VB-CB pairs, the π-like interaction between these orbitals corroborates Wheeler et al.'s conclusion that for metals with a low *d* electron count, PJTE mixing of the metal d_π and X p_π orbitals favours asymmetric X-M-X bridges[37].

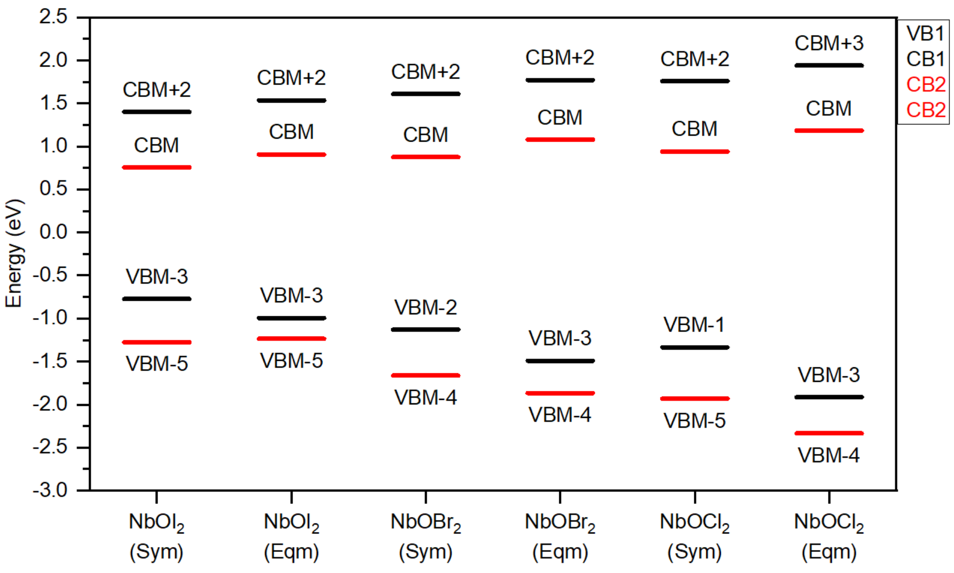

Fig. S21 | Valence and conduction band pairs involved in inducing the symmetry-breaking distortion though the PJTE. Band indices are provided at the Gamma point.

Supplementary Note 4: Strain-assisted Ferroelectric Switching

As discussed in the main text, the magnitude of the applied electric field can be much reduced if one applies compressive strain to the materials. This effect is especially large for $NbOI_2$ which has the largest piezoelectric effect.

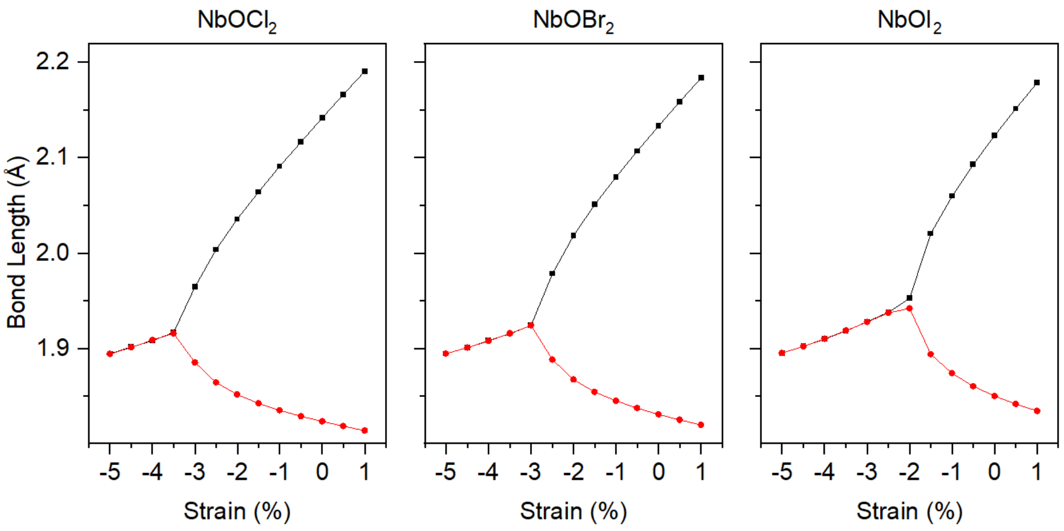

Fig. S22 | Effect of strain on the Nb-O bond lengths in $NbOX_2$. The black and red lines denote the longer and shorter Nb-O bonds respectively. A centred symmetric structure is obtained when compressive strain of over -3.5%, -3.0% and -2.5% along the x-direction is applied to $NbOCl_2$, $NbOBr_2$ and $NbOI_2$ respectively.

The large response of Nb atoms in response to strain in the x-direction also enables both the magnitude of polarization and the ferroelectric switching barriers to be controlled by strain. A modest compressive strain of -2.5% in $NbOI_2$ results in a centred symmetric structure (**Supplementary Fig. S22**), allowing the polarization direction to be set with an electric field of small magnitude. The polarization magnitude can then be enhanced by applying tensile strain.

III. High Throughput Calculation Results

Table S15 | Table of quantities obtained from high throughput calculation workflow. Formula Pretty is the human readable chemical formula of the material. **S.G. No.** is the space group number of the material. Non-ferroelectric **point groups**[38] are marked in bold. **Sheet plane vector direction** presents the direction of the 2D sheet's normal vector. **Height** presents the height (Å) of the unit cell. **Layer thickness** is the distance (Å), perpendicular to the 2D sheet, between the topmost and bottom most atoms. **Band gap** is in the unit of (eV). **Max abs piezo** is the magnitude of the largest piezoelectric stress tensor element in $C\ m^{-2}$. **Max abs sheet piezo** is the magnitude of the largest piezoelectric stress tensor element in $10^{-10}\ C\ m^{-1}$. **Max piezo index** is the index of the piezoelectric stress tensor element that has the largest magnitude. **Oop piezo** is a boolean reflecting if the material has a piezoelectric stress tensor element corresponding to the out-of-plane direction that has a magnitude larger than 0.005 $C\ m^{-2}$. **Max abs oop piezo**, **max abs sheet oop piezo** and **max oop piezo index** are out-of-plane counterparts of Max abs piezo, max abs sheet piezo and max piezo index respectively.

formula pretty	2DMatpedia ID	S.G. No.	point group	sheet plane vector direction	height	layer thickness	band gap	max abs piezo	max abs sheet piezo	max piezo index	oop piezo	max abs oop piezo	max abs sheet oop piezo	max oop piezo index
SbF_3	2dm-3709	31	mm2	z	23.009	2.882	4.441	TRUE	2.002	46.072	yxy	FALSE		
NbI_2O	2dm-4281	25	mm2	z	24.170	4.603	0.971	TRUE	1.279	30.922	xxx	FALSE		
$NbBr_2O$	2dm-4734	25	mm2	z	23.866	4.219	0.917	TRUE	1.103	26.329	xxx	FALSE		
$NbCl_2O$	2dm-3054	25	mm2	z	23.541	3.915	0.922	TRUE	1.061	24.974	xxx	FALSE		
$Mo(BrO)_2$	2dm-3188	26	mm2	z	24.536	4.862	1.631	TRUE	0.678	16.626	yyy	FALSE		
GeSe	2dm-4478	31	mm2	z	23.325	2.599	1.222	TRUE	0.526	12.270	yyy	TRUE	0.067	1.557
$SbAsO_3$	2dm-3790	7	m	z	22.588	4.178	4.040	TRUE	0.469	10.594	xxx	TRUE	0.010	0.232
$InCu(PSe_3)_2$	2dm-3689	149	**32**	z	23.339	3.424	0.503	TRUE	0.435	10.141	xxy	FALSE		
$V_2Pb_3O_8$	2dm-3693	5	2	z	23.936	4.927	3.283	TRUE	0.399	9.539	yyy	TRUE	0.056	1.330
$Zn(BH_4)_2$	2dm-4877	26	mm2	z	26.663	6.255	4.499	TRUE	0.351	9.361	xxy	FALSE		
$InSnCl_3$	2dm-4964	8	m	z	21.349	2.516	2.959	TRUE	0.344	7.349	xxx	FALSE		
Sn_2IF_3	2dm-3802	59	**mmm**	z	24.570	6.755	3.092	TRUE	0.293	7.203	yzx	FALSE		
$NbTlBr_4O$	2dm-3972	25	mm2	z	22.931	3.631	1.436	TRUE	0.293	6.708	xxx	FALSE		
$SbTeClO_3$	2dm-4969	31	mm2	z	23.441	4.092	3.210	TRUE	0.215	5.037	yxx	TRUE	0.011	0.260
As_2O_3	2dm-3779	7	m	z	22.204	3.958	4.294	TRUE	0.216	4.802	xxx	TRUE	0.011	0.246
Te_2Mo	2dm-5370	187	**-6m2**	z	23.824	3.617	1.160	TRUE	0.194	4.617	xxy	FALSE		
SrH_2O_3	2dm-4166	26	mm2	z	20.896	3.923	3.054	TRUE	0.216	4.515	yyy	TRUE	0.006	0.121

$TaPbF_7$	2dm-5388	6	m	z	23.050	3.270	5.255	TRUE	0.187	4.309	yyy	FALSE		
BaH_2O_3	2dm-4554	26	mm2	z	25.441	3.901	3.047	TRUE	0.166	4.227	yyy	TRUE	0.006	0.148
ZnH_2SeO_4	2dm-3789	7	m	z	23.395	3.932	4.458	TRUE	0.177	4.131	xyy	FALSE		
$MoSe_2$	2dm-3409	187	**-6m2**	z	23.506	3.339	1.450	TRUE	0.163	3.839	xxy	FALSE		
MoS_2	2dm-3150	187	**-6m2**	z	23.332	3.122	1.722	TRUE	0.159	3.719	yyy	FALSE		
$TlBS_3$	2dm-3554	7	m	z	23.128	3.691	2.180	TRUE	0.153	3.537	yyy	TRUE	0.008	0.193
Hg_3SeO_6	2dm-5414	8	m	z	21.942	2.188	1.095	TRUE	0.156	3.431	yyy	TRUE	0.028	0.609
$InGaS_3$	2dm-3760	31	mm2	z	26.993	7.216	1.986	TRUE	0.124	3.349	zyz	TRUE	0.124	3.349
Te_2W	2dm-3050	187	**-6m2**	z	23.749	3.630	1.193	TRUE	0.134	3.187	yxx	FALSE		
CuHgSeCl	2dm-5612	26	mm2	z	28.830	3.980	0.798	TRUE	0.110	3.181	yyy	TRUE	0.008	0.229
As_2Se_3	2dm-4755	31	mm2	x	22.455	2.906	1.770	TRUE	0.141	3.158	yyy	FALSE		
WSe_2	2dm-3594	187	**-6m2**	z	23.485	3.360	1.543	TRUE	0.111	2.596	xxy	FALSE		
WS_2	2dm-3749	187	**-6m2**	z	23.220	3.141	1.805	TRUE	0.109	2.541	yxx	FALSE		
$AgBi(PSe_3)_2$	2dm-5567	143	3	z	23.398	3.582	1.442	TRUE	0.108	2.523	yxx	TRUE	0.006	0.150
GaTeCl	2dm-3523	31	mm2	z	24.660	5.327	2.296	TRUE	0.098	2.429	zyz	TRUE	0.098	2.429
SrH_4O_3	2dm-3672	26	mm2	z	24.443	6.567	4.365	TRUE	0.094	2.310	yyy	TRUE	0.010	0.235
$SiAs_2$	2dm-5490	26	mm2	z	25.682	6.012	1.443	TRUE	0.089	2.283	yyy	TRUE	0.006	0.154
$HfGeTe_4$	2dm-4668	31	mm2	z	27.410	7.433	0.769	TRUE	0.078	2.128	yyy	FALSE		
$GeAs_2$	2dm-3619	26	mm2	z	25.907	6.056	1.237	TRUE	0.080	2.073	yyy	TRUE	0.007	0.170
$CuBi(PSe_3)_2$	2dm-4194	143	3	z	23.507	3.621	1.245	TRUE	0.085	1.994	yxx	TRUE	0.007	0.153
P_2O_5	2dm-3519	31	mm2	z	25.403	5.462	5.080	TRUE	0.077	1.967	yyy	TRUE	0.012	0.311
$HgINO_3$	2dm-3984	26	mm2	z	21.792	3.692	2.177	TRUE	0.088	1.916	yxx	TRUE	0.007	0.142
BiTeI	2dm-3590	156	3m	z	23.653	3.773	1.512	TRUE	0.080	1.901	yyy	TRUE	0.007	0.177
$AlHO_2$	2dm-4724	31	mm2	z	25.476	5.350	4.166	TRUE	0.073	1.872	yyy	TRUE	0.014	0.354
GaS	2dm-3608	187	**-6m2**	z	24.524	4.643	2.396	TRUE	0.076	1.867	yyy	FALSE		
Ag_3SI	2dm-5200	4	2	z	29.013	9.639	0.547	TRUE	0.063	1.839	yyy	TRUE	0.012	0.339
$NaTaCl_6$	2dm-3691	4	2	z	26.982	9.434	2.919	TRUE	0.068	1.821	yyy	TRUE	0.008	0.206
$ZrGeTe_4$	2dm-3329	31	mm2	z	27.565	7.481	0.689	TRUE	0.066	1.821	yyy	TRUE	0.010	0.272
GaSe	2dm-3530	187	**-6m2**	z	24.648	4.822	1.790	TRUE	0.073	1.789	yyy	FALSE		
$InAg(PSe_3)_2$	2dm-3598	149	**32**	z	23.469	3.563	0.901	TRUE	0.076	1.785	yxx	FALSE		
$AsPO_4$	2dm-5240	31	mm2	z	25.424	4.837	4.364	TRUE	0.068	1.726	yxx	FALSE		
As_2S_3	2dm-4821	31	mm2	x	21.848	2.594	2.299	TRUE	0.079	1.723	yyy	FALSE		
UCO_5	2dm-4524	25	mm2	z	23.359	3.571	2.294	TRUE	0.070	1.638	xxx	FALSE		
SiP_2	2dm-4912	26	mm2	z	25.623	5.592	1.550	TRUE	0.063	1.609	yyy	FALSE		
$VAg(PSe_3)_2$	2dm-4708	5	2	z	23.438	3.548	0.330	TRUE	0.066	1.556	xyy	FALSE		
$Cd(IO_3)_2$	2dm-3721	4	2	z	26.236	7.374	3.531	TRUE	0.059	1.546	zzx	TRUE	0.059	1.546
$B_2S_2O_9$	2dm-3130	5	2	z	26.564	7.282	6.834	TRUE	0.058	1.543	xxx	TRUE	0.048	1.273
$Ca(AuF_6)_2$	2dm-3472	115	**-42m**	z	23.867	7.078	1.639	TRUE	0.063	1.506	xzx	FALSE		
BN	2dm-4991	187	**-6m2**	z	19.935	0.000	4.711	TRUE	0.073	1.462	xxy	FALSE		
$Mn(CuCl_2)_2$	2dm-5365	25	mm2	z	20.365	2.840	0.926	TRUE	0.071	1.455	xzx	TRUE	0.010	0.213
$GaAg(PSe_3)_2$	2dm-4552	149	**32**	z	23.369	3.462	0.938	TRUE	0.061	1.418	yxx	FALSE		
$Hg_2P_2S_7$	2dm-3704	5	2	z	24.357	6.217	2.111	TRUE	0.056	1.365	xyy	FALSE		
BiTeBr	2dm-4356	156	3m	z	23.305	3.566	1.607	TRUE	0.053	1.241	xxy	FALSE		
Hg_3AsS_4Cl	2dm-4753	156	3m	z	22.449	3.066	2.125	TRUE	0.055	1.233	xxy	FALSE		
$HfFeCl_6$	2dm-5854	5	2	z	21.980	2.939	0.241	TRUE	0.053	1.174	xyy	FALSE		
Nb_3TeI_7	2dm-3841	156	3m	z	23.847	3.956	0.611	TRUE	0.040	0.945	xxy	TRUE	0.009	0.206
Hg_3AsSe_4Br	2dm-4674	156	3m	z	22.795	3.338	1.803	TRUE	0.040	0.922	yyy	FALSE		
$ScAg(PS_3)_2$	2dm-5836	149	**32**	z	23.162	3.358	2.045	TRUE	0.040	0.922	xxy	FALSE		

$InAg(PS_3)_2$	2dm-3602	149	**32**	z	23.185	3.402	1.365	TRUE	0.040	0.922	yxx	FALSE		
Ta_3SeI_7	2dm-5470	156	3m	z	23.628	3.772	0.700	TRUE	0.037	0.886	yxx	TRUE	0.010	0.240
Nb_3SBr_7	2dm-3765	156	3m	z	23.159	3.451	0.801	TRUE	0.037	0.865	xxy	TRUE	0.011	0.245
Ta_3TeI_7	2dm-5496	156	3m	z	23.831	3.998	0.667	TRUE	0.036	0.863	yxx	TRUE	0.007	0.169
InSe	2dm-3459	187	**-6m2**	z	24.994	5.381	1.386	TRUE	0.034	0.848	yyy	FALSE		
$LaBr_2$	2dm-5867	187	**-6m2**	z	23.350	3.828	0.625	TRUE	0.036	0.829	yxx	FALSE		
BiTeCl	2dm-3732	156	3m	z	23.181	3.375	1.778	TRUE	0.035	0.822	yyz	FALSE		
Nb_3TeCl_7	2dm-3785	156	3m	z	23.374	3.723	0.772	TRUE	0.032	0.751	xxy	FALSE		
Ta_3SBr_7	2dm-5348	156	3m	z	23.177	3.468	0.872	TRUE	0.032	0.745	yyy	TRUE	0.009	0.206
CuO_2F	2dm-4542	17	**222**	z	19.541	1.969	1.044	TRUE	0.038	0.736	yzx	FALSE		
$CdTeMoO_6$	2dm-4591	113	**-42m**	z	27.457	7.962	3.555	TRUE	0.026	0.703	yzx	TRUE	0.009	0.244
CaHClO	2dm-4557	156	3m	z	23.101	3.583	3.598	TRUE	0.028	0.650	yyz	TRUE	0.014	0.334
$ZrCl_2$	2dm-3706	187	**-6m2**	z	23.475	3.427	1.028	TRUE	0.027	0.639	xxy	FALSE		
$MnTeMoO_6$	2dm-3666	18	**222**	z	27.418	7.809	2.517	TRUE	0.021	0.571	yzx	FALSE		
$Ge_3Sb_2O_9$	2dm-3499	174	**-6**	z	23.773	4.784	3.950	TRUE	0.021	0.501	yxy	FALSE		
$LaHBr_2$	2dm-4199	187	**-6m2**	z	23.774	3.900	3.767	TRUE	0.019	0.462	yxx	FALSE		
$Hg_3(BO_3)_2$	2dm-4803	189	**-6m2**	z	18.639	0.003	3.401	TRUE	0.024	0.452	xxx	FALSE		
Nb_3I_8	2dm-5497	156	3m	z	23.975	4.058	0.233	TRUE	0.017	0.418	xxy	FALSE		
H_3BrO	2dm-5037	156	3m	z	21.265	1.116	5.225	TRUE	0.018	0.386	zxx	TRUE	0.018	0.386
$TlAsO_4$	2dm-5146	111	**-42m**	z	22.060	2.087	1.492	TRUE	0.017	0.376	xyz	TRUE	0.015	0.322
$Sn(PS_3)_2$	2dm-5267	149	**32**	z	22.825	3.369	1.367	TRUE	0.016	0.365	yyy	FALSE		
Ag_2SO_4	2dm-4885	21	**222**	z	20.124	1.791	2.248	TRUE	0.016	0.317	zxy	TRUE	0.016	0.317
Ag_2SeO_4	2dm-3267	21	**222**	z	20.413	2.028	1.646	TRUE	0.015	0.311	zxy	TRUE	0.015	0.311
$TmAg(PSe_3)_2$	2dm-5578	149	**32**	z	23.395	3.598	1.839	TRUE	0.013	0.302	xxy	FALSE		
Nb_3Cl_8	2dm-5206	156	3m	z	23.184	3.497	0.246	TRUE	0.013	0.290	yyy	FALSE		
$CuSe_2Cl$	2dm-4225	17	**222**	z	22.517	2.805	0.926	TRUE	0.013	0.284	yzx	TRUE	0.007	0.154
NaHO	2dm-5304	129	**4/mmm**	z	23.838	4.605	2.837	TRUE	0.012	0.281	zzx	TRUE	0.012	0.281
AgO_2F	2dm-4445	17	**222**	z	22.089	2.098	1.062	TRUE	0.012	0.256	zxy	TRUE	0.012	0.256
$CuSe_2Br$	2dm-4942	17	**222**	z	22.518	3.126	0.933	TRUE	0.011	0.248	yzx	TRUE	0.006	0.144
Cu_2WS_4	2dm-4517	111	**-42m**	z	22.442	2.601	1.251	TRUE	0.010	0.231	xyz	TRUE	0.006	0.125
$LiBH_4$	2dm-3894	156	3m	z	21.314	1.664	6.282	TRUE	0.010	0.222	zxx	TRUE	0.010	0.222
Cu_2SO_4	2dm-3586	21	**222**	z	20.331	1.786	2.120	TRUE	0.010	0.208	zxy	TRUE	0.010	0.208
Cu_2WSe_4	2dm-3107	111	**-42m**	z	22.514	2.839	1.233	TRUE	0.009	0.207	xyz	TRUE	0.008	0.181
LiH_2N	2dm-3071	113	**-42m**	z	23.764	3.461	3.202	TRUE	0.008	0.194	xyz	FALSE		
$ScAg(PSe_3)_2$	2dm-5821	149	**32**	z	23.376	3.545	1.694	TRUE	0.008	0.188	yyy	FALSE		
$CuTe_2Br$	2dm-5156	17	**222**	z	22.242	3.044	0.912	TRUE	0.008	0.183	yzx	TRUE	0.005	0.120
$CuTe_2Cl$	2dm-4859	17	**222**	z	21.769	2.749	0.886	TRUE	0.008	0.180	yzx	TRUE	0.005	0.112
$AuBrO_2$	2dm-4476	17	**222**	z	21.615	3.040	0.963	TRUE	0.008	0.177	xyz	FALSE		
AuO_2F	2dm-4797	17	**222**	z	20.562	2.153	1.350	TRUE	0.008	0.169	yzx	FALSE		
$AuClO_2$	2dm-3945	17	**222**	z	22.425	2.790	1.088	TRUE	0.007	0.152	xyz	FALSE		
$CuTe_2I$	2dm-5239	17	**222**	z	22.415	3.385	0.960	TRUE	0.006	0.144	yzx	TRUE	0.006	0.143
$ErAg(PSe_3)_2$	2dm-5480	149	**32**	z	23.481	3.602	1.819	TRUE	0.006	0.140	xxy	FALSE		
$ZnCl_2$	2dm-4713	115	**-42m**	z	22.100	2.724	4.247	TRUE	0.004	0.088	zxx	FALSE		
Li_2WS_4	2dm-5501	111	**-42m**	z	22.171	2.486	1.933	TRUE	0.003	0.058	yzx	FALSE		

IV. References

1. Gao W, Chelikowsky JR. Prediction of Intrinsic Ferroelectricity and Large Piezoelectricity in Monolayer Arsenic Chalcogenides. *Nano Letters* **20**, 8346-8352 (2020).
2. Wang H, Qian X. Two-dimensional multiferroics in monolayer group IV monochalcogenides. *2D Materials* **4**, 015042 (2017).
3. Fei R, Li W, Li J, Yang L. Giant piezoelectricity of monolayer group IV monochalcogenides: SnSe, SnS, GeSe, and GeS. *Applied Physics Letters* **107**, 173104 (2015).
4. Duerloo K-AN, Ong MT, Reed EJ. Intrinsic Piezoelectricity in Two-Dimensional Materials. *The Journal of Physical Chemistry Letters* **3**, 2871-2876 (2012).
5. Li W, Li J. Piezoelectricity in two-dimensional group-III monochalcogenides. *Nano Research* **8**, 3796-3802 (2015).
6. You L*, et al.* Origin of giant negative piezoelectricity in a layered van der Waals ferroelectric. *Science Advances* **5**, eaav3780 (2019).
7. Giannozzi P*, et al.* QUANTUM ESPRESSO: a modular and open-source software project for quantum simulations of materials. *Journal of Physics: Condensed Matter* **21**, 395502 (2009).
8. Giannozzi P*, et al.* Advanced capabilities for materials modelling with Quantum ESPRESSO. *Journal of Physics: Condensed Matter* **29**, 465901 (2017).
9. Giannozzi P*, et al.* Quantum ESPRESSO toward the exascale. *The Journal of Chemical Physics* **152**, 154105 (2020).
10. Blöchl PE. Projector augmented-wave method. *Physical Review B* **50**, 17953-17979 (1994).
11. Perdew JP, Burke K, Ernzerhof M. Generalized Gradient Approximation Made Simple. *Physical Review Letters* **77**, 3865-3868 (1996).
12. Kresse G, Furthmüller J. Efficient iterative schemes for ab initio total-energy calculations using a plane-wave basis set. *Physical Review B* **54**, 11169-11186 (1996).
13. Monkhorst HJ, Pack JD. Special points for Brillouin-zone integrations. *Physical Review B* **13**, 5188-5192 (1976).
14. Zhu H*, et al.* Observation of piezoelectricity in free-standing monolayer MoS2. *Nature Nanotechnology* **10**, 151-155 (2015).
15. Ares P*, et al.* Piezoelectricity in Monolayer Hexagonal Boron Nitride. *Advanced Materials* **32**, (2020).
16. Lu A-Y*, et al.* Janus monolayers of transition metal dichalcogenides. *Nature Nanotechnology* **12**, 744-749 (2017).
17. Sharma P*, et al.* A room-temperature ferroelectric semimetal. *Science Advances* **5**, eaax5080 (2019).
18. Zelisko M*, et al.* Anomalous piezoelectricity in two-dimensional graphene nitride nanosheets. *Nature Communications* **5**, 4284 (2014).
19. Wang X*, et al.* Subatomic deformation driven by vertical piezoelectricity from CdS ultrathin films. *Science Advances* **2**, e1600209 (2016).
20. Li P, Zhang Z, Shen W, Hu C, Shen W, Zhang D. A self-powered 2D-material sensor unit driven by a SnSe piezoelectric nanogenerator. *Journal of Materials Chemistry A* **9**, 4716-4723 (2021).
21. Zhao M-H, Wang Z-L, Mao SX. Piezoelectric Characterization of Individual Zinc Oxide Nanobelt Probed by Piezoresponse Force Microscope. *Nano Letters* **4**, 587-590 (2004).
22. Beck J, Kusterer C. Crystal Structure of NbOBr2. *Zeitschrift für anorganische und allgemeine Chemie* **632**, 2193-2194 (2006).

23. Rijnsdorp J, Jellinek F. The crystal structure of niobium oxide diiodide NbOI2. *Journal of the Less Common Metals* **61**, 79-82 (1978).
24. Henkelman G, Arnaldsson A, Jónsson H. A fast and robust algorithm for Bader decomposition of charge density. *Computational Materials Science* **36**, 354-360 (2006).
25. Tang W, Sanville E, Henkelman G. A grid-based Bader analysis algorithm without lattice bias. *Journal of Physics: Condensed Matter* **21**, 084204 (2009).
26. Yu M, Trinkle DR. Accurate and efficient algorithm for Bader charge integration. *The Journal of Chemical Physics* **134**, 064111 (2011).
27. Sanville E, Kenny SD, Smith R, Henkelman G. Improved grid-based algorithm for Bader charge allocation. *Journal of Computational Chemistry* **28**, 899-908 (2007).
28. Hughbanks T, Hoffmann R. Chains of trans-edge-sharing molybdenum octahedra: metal-metal bonding in extended systems. *Journal of the American Chemical Society* **105**, 3528-3537 (1983).
29. Kochervinskii VV. Piezoelectricity in crystallizing ferroelectric polymers: Poly(vinylidene fluoride) and its copolymers (A review). *Crystallography Reports* **48**, 649-675 (2003).
30. Wu X, Vanderbilt D, Hamann DR. Systematic treatment of displacements, strains, and electric fields in density-functional perturbation theory. *Physical Review B* **72**, 035105 (2005).
31. Baroni S, de Gironcoli S, Dal Corso A, Giannozzi P. Phonons and related crystal properties from density-functional perturbation theory. *Reviews of Modern Physics* **73**, 515-562 (2001).
32. Gonze X. Adiabatic density-functional perturbation theory. *Physical Review A* **52**, 1096-1114 (1995).
33. Choudhary K, Cheon G, Reed E, Tavazza F. Elastic properties of bulk and low-dimensional materials using van der Waals density functional. *Physical Review B* **98**, 014107 (2018).
34. Higashitarumizu N*, et al.* Purely in-plane ferroelectricity in monolayer SnS at room temperature. *Nature Communications* **11**, 2428 (2020).
35. Kwon KC*, et al.* In-Plane Ferroelectric Tin Monosulfide and Its Application in a Ferroelectric Analog Synaptic Device. *ACS Nano* **14**, 7628-7638 (2020).
36. Bersuker IB. Pseudo-Jahn–Teller Effect—A Two-State Paradigm in Formation, Deformation, and Transformation of Molecular Systems and Solids. *Chemical Reviews* **113**, 1351-1390 (2013).
37. Wheeler RA, Whangbo MH, Hughbanks T, Hoffmann R, Burdett JK, Albright TA. Symmetric vs. asymmetric linear M-X-M linkages in molecules, polymers, and extended networks. *Journal of the American Chemical Society* **108**, 2222-2236 (1986).
38. Smidt TE, Mack SA, Reyes-Lillo SE, Jain A, Neaton JB. An automatically curated first-principles database of ferroelectrics. *Scientific Data* **7**, 72 (2020).
